# Inhibitors of Calcium Oxalate Crystallization for the Treatment of Oxalate Nephropathies

**DOI:** 10.1002/advs.201903337

**Published:** 2020-02-27

**Authors:** Anna Kletzmayr, Shrikant R. Mulay, Manga Motrapu, Zhi Luo, Hans‐Joachim Anders, Mattias E. Ivarsson, Jean‐Christophe Leroux

**Affiliations:** ^1^ Institute of Pharmaceutical Sciences Department of Chemistry and Applied Biosciences ETH Zurich 8093 Zurich Switzerland; ^2^ Division of Nephrology Department of Medicine IV University Hospital LMU Munich 80336 Munich Germany; ^3^ Inositec Inc. 8005 Zurich Switzerland

**Keywords:** calcium oxalate crystallization inhibitors, chronic kidney disease, image‐based drug screening, kidney calcification, kidney stones

## Abstract

Calcium oxalate (CaOx) crystal‐induced nephropathies comprise a range of kidney disorders, for which there are no efficient pharmacological treatments. Although CaOx crystallization inhibitors have been suggested as a therapeutic modality already decades ago, limited progress has been made in the discovery of potent molecules with efficacy in animal disease models. Herein, an image‐based machine learning approach to systematically screen chemically modified *myo*‐inositol hexakisphosphate (IP6) analogues is utilized, which enables the identification of a highly active divalent inositol phosphate molecule. To date, this is the first molecule shown to completely inhibit the crystallization process in the nanomolar range, reduce crystal–cell interactions, thereby preventing CaOx‐induced transcriptomic changes, and decrease renal CaOx deposition and kidney injury in a mouse model of hyperoxaluria. In conclusion, IP6 analogues based on such a scaffold may represent a new treatment option for CaOx nephropathies.

## Graphical abstract

Calcium oxalate (CaOx) crystallization in the kidney comprises a broad range of disorders, causing tissue calcification and kidney stone formation. Using an image‐based compound screening approach, a potent, in vivo efficacious, CaOx inhibitory molecule is identified, exhibiting a dual mode of action. First, the compound inhibits renal CaOx crystallization and second, it prevents CaOx–cell interactions and CaOx‐induced renal injury.

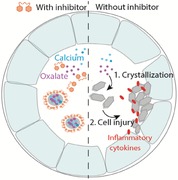

## 1 Introduction

Calcium oxalate (CaOx) crystals in the kidney elicit renal injury, which in turn leads to the development of a range of kidney disorders, such as nephrocalcinosis, kidney stones, and acute or chronic kidney disease (CKD).^[^ 1, 2, 3, 4 ^]^ CaOx crystallization in the kidney tubules is caused by elevated urinary oxalate levels occurring in, for example, genetic disorders, such as primary hyperoxaluria or genetic forms of renal tubular acidosis,^[^ 1, 3, 5 ^]^ or in diseases associated with malabsorption (i.e., enteric hyperoxaluria).^[^ 2, 6 ^]^ It is suggested that upon formation, CaOx crystals can adhere directly to the renal epithelium^[^ 7, 8, 9 ^]^ or to hydroxyapatite deposits, so‐called Randall's plaques, forming a nidus for growth and aggregation with urinary proteins leading to kidney stones.^[^ 2, 10 ^]^ Further, CaOx crystal deposition in the kidney tubules elicits cytotoxicity, inflammation of the tissue, and necrosis contributing to kidney injury.^[^ 11, 12, 13, 14 ^]^

To date, treatment options for CaOx nephropathies are extremely limited, mostly comprising supportive rather than curative approaches.^[^ 1, 2, 4, 5 ^]^ Existing kidney stones can be surgically removed.^[^ 4 ^]^ Treatment with urinary alkalizing agents, such as citrate, has shown beneficial effects in clinical studies due to the reduction of CaOx crystallization at increased pH.^[^ 1, 2, 4, 5, 15 ^]^ However, upon chronic or repeated exposure to CaOx crystals, the current procedures can merely delay the progression towards CKD and, ultimately, end stage kidney disease.^[^ 1, 4, 16 ^]^ At this stage, organ transplantation presents the last resort for patients.^[^ 1, 16 ^]^ Considering the heterogeneity of CaOx nephropathies, a strategy to reduce renal CaOx crystallization and crystal–cell interactions independent of the etiology could be a favorable therapeutic modality applicable to a wide range of indications.

CaOx crystallization inhibitors have already been proposed as a general therapeutic modality in the 1960s, but bridging in vitro to in vivo efficacy has proven to be very challenging.^[^ 17, 18 ^]^ A broad array of candidate molecules has been explored ranging from various small molecules, such as citrate derivatives or polyphosphates, to macromolecules, including peptides and synthetic polymers with carboxylic acid, sulfate, hydroxyl, and/or phosphate groups.^[^ 17, 18, 19, 20, 21, 22, 23, 24, 25 ^]^ In recent years, the majority of studies focused on the effects of endogenous urinary CaOx inhibitors, such as osteopontin, and synthetic peptide mimetics rich in aspartic and glutamic acid residues.^[^ 21, 23, 24, 25 ^]^ Both importance of negative charge of the inhibitor, as well as the specific amino acid sequence on the molecules' capability to modify crystal growth were emphasized by these studies.^[^ 20, 21, 23, 25, 26 ^]^ Nonetheless, most reports have focused on the tested molecules' effects on crystal morphology, while their potency to completely prevent crystallization or inhibit crystal–cell interactions was neglected, and evidence of their activity in animal disease models was lacking.^[^ 18, 20, 21, 22, 25 ^]^ Accordingly, the feasibility of developing clinically viable CaOx crystallization inhibitors as a therapeutic modality in CaOx nephropathies was questioned in recent years.^[^ 17 ^]^

In this study, we aimed at designing novel CaOx inhibitors by investigating the effect of chemical modifications of *myo*‐inositol hexakisphosphate (IP6) on its function to inhibit CaOx crystallization and CaOx–cell interactions. IP6, a highly negatively charged small molecule found ubiquitously in eukaryotic cells, has been shown to be an effective inhibitor of calcium phosphate crystallization.^[^ 27, 28 ^]^ Substituting phosphate groups with different anionic groups or oligo‐ethylene glycol (OEG) chains led to the development of molecules with increased stability against enzymatic degradation, tunable calcium binding properties,^[^ 29 ^]^ and enhanced inhibition of calcium phosphate crystallization.^[^ 30 ^]^ Thus, we hypothesized that the described molecules might provide a promising starting point for the engineering of potent renal CaOx inhibitors.

Herein, we present the development of a new class of highly efficacious IP6‐based inhibitors of renal CaOx crystallization. The combination of electron microscopy with a machine learning based analysis of light microscopy images allowed us to closely follow crystallization dynamics in a rapid manner, thereby unveiling the mechanism of inhibition. The systematic screening of a series of IP6 analogues led to the identification of new active compounds with nanomolar efficacy. Interesting insights into the pathomechanism of CaOx nephropathies and inhibitory pathways thereof were further gained by RNA sequencing studies on the interplay of CaOx crystals with renal epithelial cells. Importantly, activity of our lead compound was confirmed in a mouse disease model.

## 2 Results

### 2.1 Image‐Based Screening of CaOx Crystallization Dynamics

**Figure 1:**
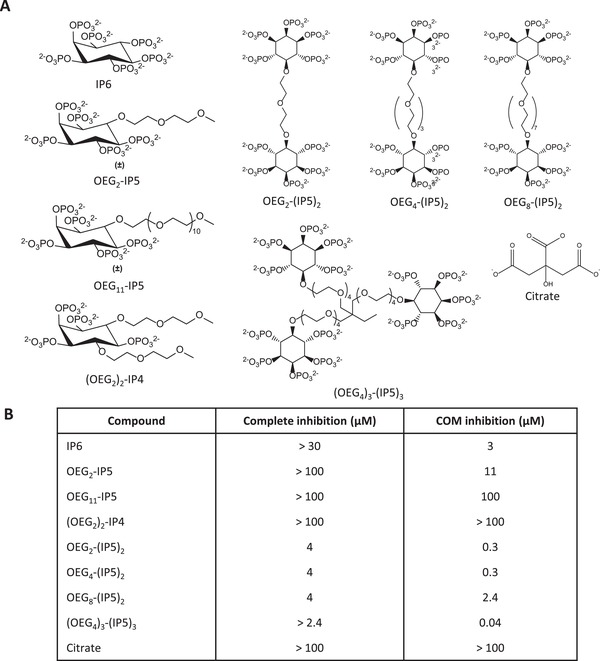
Overview of efficacy of tested compounds to inhibit CaOx crystallization in human urine. A) Structures of the compounds tested in the CaOx screening assay. B) Minimal mean concentrations required for complete, COM and COD, inhibition of crystallization (total area < 5% of total area control), and inhibition of COM‐only crystallization in the CaOx screening assay (COM total area < 5% of total area control) are given (*N* = 3).

While IP6 is a known inhibitor of calcium phosphate crystallization,^[^ 27, 28 ^]^ little is known about the inhibitory properties of IP6 on CaOx crystallization.^[^ 31 ^]^ Thus, in a first step we investigated the effects of IP6 on CaOx crystallization in human urine, as well as the impact of chemical modifications on the in vitro CaOx growth inhibition efficacy of the molecule (**Figure** **1**).

**Figure 2:**
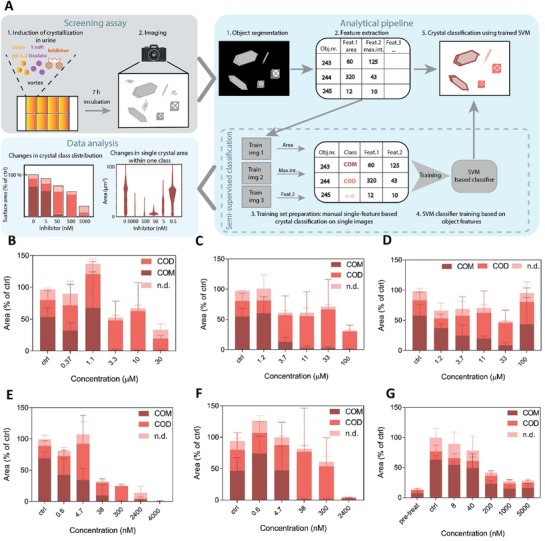
Changes in the CaOx crystallization pattern induced by IP6 analogues. A) Outline of the CaOx screening assay. Effects of B) IP6, C) OEG_2_‐IP5, D) (OEG_2_)_2_‐IP4, E) OEG_4_‐(IP5)_2_, and F) (OEG_4_)_3_‐(IP5)_3_ on CaOx bulk crystallization in human urine spiked with 1 mm NaOx were assessed by light microscopy at *t* = 7 h. G) A modified version of the CaOx screening assay was performed, wherein OEG_4_‐(IP5)_2_ was added after CaOx crystal formation at *t* = 1.5 h. Effects on bulk crystallization showed reduction in COM total area by increasing concentrations of OEG_4_‐(IP5)_2_. The mean total area/field of view + SD for the respective crystal type normalized to the control (without inhibitor) is plotted (*N* = 3; COM—CaOx monohydrate, COD—CaOx dihydrate, n.d.—not defined).

To assess CaOx crystallization in a physiologically relevant setting, human urine was spiked with 1 mm sodium oxalate (NaOx), representing the upper concentrations reached within the renal tubules.^[^ 32 ^]^ Changes in the crystallization pattern with respect to the size and proportion of different CaOx hydrate crystal forms were monitored by light microscopy combined with a semi‐supervised image analysis approach. Similar to a previous report,^[^ 33 ^]^ images were segmented into single crystals using classical computer vision techniques and shape, intensity, and texture features for each crystal were extracted. However, in contrast to the previous study,^[^ 33 ^]^ no trace fluorescent label was used to detect the crystals in the present work, as brightfield images were found to be sufficient for image analysis. Thereby, we avoided the risk that an added component (i.e., the fluorescent label) could alter the CaOx crystallization dynamics. A training dataset was created by manually adjusting single features to distinguish the different crystal types on single images, which served as input to train a support vector machine (SVM) classifier. Subsequently, the trained SVM was used to classify crystal polymorphs on testing sample images (**Figure** **2**A; Figure S1, Supporting Information; see Experimental Section for more details). Kinetic analysis of oxalate‐spiked human urine showed first the appearance of CaOx dihydrate (COD) crystals, followed by CaOx monohydrate (COM) crystals over the time course of 24 h (Figure S2, Supporting Information). The total number and the total area for each crystal type per field of view were calculated, and normalized to the 24 h time point for each independent experiment. COM crystals were more abundant but smaller in size compared to COD, with an approximate length of 10 and 20 µm, respectively.

### 2.2 CaOx Inhibitory Efficacy Is Dependent on Phosphate Group Number

Addition of IP6 to the reaction mixture led to selective inhibition of COM crystals at concentrations above 3 µm (Figure 1B), while COD crystals remained similar to the positive control conditions (without inhibitor) in terms of total COD area at *t* = 7 h (Figure 2B). The comparison of inhibitors mainly focused on COM inhibitory concentrations and complete (COM + COD) inhibitory concentrations because the crystallization process, in particular the nucleation step, is variable by nature in terms of the extent of crystallization that occurs once it is triggered. However, we found that COM inhibitory and complete (COM and COD) inhibitory concentrations were highly reproducible across the experiments. Substitution of phosphate groups of IP6 with one and two OEG segments led to an increasing loss of COM inhibitory activity (Figures 1 and 2C,D), as shown by COM inhibitory concentrations of 11 µm for OEG_2_‐IP5 and above the tested concentrations (>100 µm) for (OEG_2_)_2_‐IP4 (Figure 1B), indicating the importance of the negatively charged phosphate group on inhibitory function. Size distribution analysis of COM crystals formed in the presence of OEG_2_‐IP5, together with visual examination of the images, further showed a dose‐dependent reduction in the crystal size, thus indicating a COM growth inhibitory effect (Figures S3A and S4, Supporting Information). Increasing length of the OEG chain from two to eleven ethylene glycol repeat units appeared to have a negligible impact on crystallization inhibition, as supported by the same COM inhibitory concentration for OEG_2_‐IP5 and OEG_11_‐IP5 (Figure 2C; Figure S5A, Supporting Information). Citrate, which is used in the clinic to treat renal CaOx crystallization as one of the few available treatment options,^[^ 1, 2, 4, 5, 15 ^]^ did not lead to any detectable CaOx inhibition up to 100 µm in our assay (Figure S5B, Supporting Information). This finding is not surprising considering that in vivo citrate likely reduces CaOx crystallization by complex alterations of urine solute excretion that result in urine alkalinization,^[^ 34 ^]^ which cannot be measured in this in vitro assay.

### 2.3 Novel Multivalent IP5 Molecules Show a Drastically Increased CaOx Inhibition Efficacy

Because only partial CaOx inhibition in the micromolar range was achieved by OEG_2_‐IP5, we sought to design more potent IP6 analogues. The observed importance of the number of negatively charged groups on inhibitory efficacy led to the synthesis of di‐ and trivalent IP5 molecules. The new molecules showed a drastically increased COM inhibition efficacy in the nanomolar range, as well as complete inhibition of CaOx crystallization (i.e., COM and COD) in the low micromolar range (Figures 1B and 2E,F; Figure S3B, Supporting Information). Increasing length of the EG linker from two to eight EG repeat units (Figure 1B; Figure S5C,D, Supporting Information) did not lead to major changes in activity between the molecules. The difference in efficacy between di‐ and trivalent IP5 was relatively small, with COM inhibition concentrations of 40 and 300 nm for the tri‐ and divalent molecules, respectively (Figures 1 and 2E,F). For further studies OEG_4_‐(IP5)_2_ was chosen over (OEG_4_)_3_‐(IP5)_3_, given its good activity and simpler synthesis.

To further investigate the activity of OEG_4_‐(IP5)_2_ on already existing crystals in the renal tubules, an adapted version of the CaOx screening assay described above was employed. In this assay, the compound was added directly to the imaging well after CaOx formation at 1.5 h and incubated for further 5.5 h. A dose‐dependent reduction in total COM area was measured, showing the stabilization of small COM crystals starting at 1 µm OEG_4_‐(IP5)_2_ (Figure 2G). Size distribution analysis of COM particles confirmed that the effect can be ascribed to an inhibition of further growth of small COM crystals (Figure S6, Supporting Information). No effects on COD crystallization were observed, likely because no further COD growth takes place after 1.5 h. Measurements of free calcium concentrations in urine upon addition of OEG_4_‐(IP5)_2_ showed calcium chelation only at concentrations of 500 µm or higher (Figure S7, Supporting Information). Thus, it can be concluded that the CaOx crystal growth inhibition is not simply an indirect consequence of lowering free calcium but caused by direct crystal–inhibitor interactions.

### 2.4 OEG_4_‐(IP5)_2_ Inhibits CaOx Crystallization by Early‐Stage Precursor Stabilization

**Figure 3:**
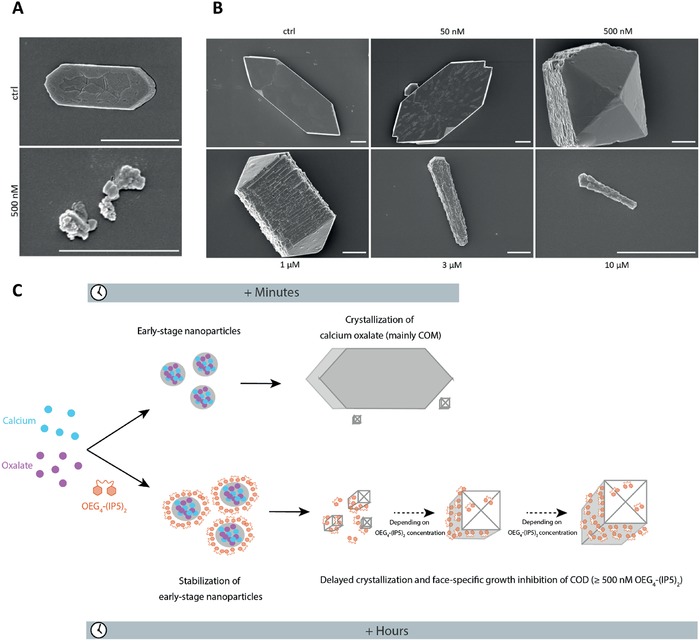
OEG_4_‐(IP5)_2_ stabilizes early precursor particles and leads to delayed and decelerated COD growth. A) CaOx crystallization in Bis‐Tris buffer (pH 6.2) with 500 nm OEG_4_‐(IP5)_2_ and without inhibitor (ctrl) was sampled at *t* = 10 min and imaged by SEM (scale bar: 1 µm). While without inhibitor predominantly COM crystals were observed, with 500 nm OEG_4_‐(IP5)_2_ round nanoparticles without a distinct COM or COD shape were found. B) Dose‐dependent inhibition of CaOx crystallization by OEG_4_‐(IP5)_2_ at *t* = 7 h. Representative SEM images are shown (scale bar: 1 µm). After 7 h of incubation, in the buffer ctrl sample predominantly COM crystals around 10 µm in size are observed, similar to ≤ 50 nm OEG_4_‐(IP5)_2_ . Concentrations of ≥500 nm OEG_4_‐(IP5)_2_ resulted in COD crystallization and a dose‐dependent COD inhibition. C) Schematic drawing of the proposed mechanism of CaOx crystallization inhibition by OEG_4_‐(IP5)_2_.

While previous studies mostly focused on the effect of inhibitors on COM growth,^[^ 20, 22, 25 ^]^ our assay captured the dynamics and interplay of both COM and COD crystallization. Hence, we further investigated the mechanism of CaOx inhibition by OEG_4_‐(IP5)_2_ in a simplified Bis‐Tris buffer system. Scanning electron microscopy (SEM) experiments of early stage crystallization at *t* = 10 min revealed the presence of both, COM crystals and round nanosized particles, while with 500 nm OEG_4_‐(IP5)_2_ only nanosized particles were detected (**Figure** **3**A). Nanosized particles were still present at *t* = 1 h with 0.5–10 µm of inhibitor, in contrast to micrometer sized COM crystals being predominant in the control sample (Figure S8A, Supporting Information). These results suggest the stabilization of early stage, nanosized CaOx particles by OEG_4_‐(IP5)_2_, thereby drastically delaying crystallization.

SEM experiments further confirmed a dose‐dependent shift from mostly‐COM to mostly‐COD crystallization with increasing concentrations of OEG_4_‐(IP5)_2_ starting from 500 nm in the Bis‐Tris buffer system (Figure 3B, also see Figure 2E for comparable screening results in urine). In conjunction, face‐specific growth, possibly caused by preferential binding of the inhibitor to a certain crystal face due to its unique molecular surface arrangement,^[^ 20, 25 ^]^ was observed by an elongation of COD crystals into a needle‐like appearance with increasing concentrations of OEG_4_‐(IP5)_2_ in both the screening experiments and SEM (Figure 3B; Figure S3B, Supporting Information). Single‐crystal X‐ray diffraction of both the classic tetragonal bipyramidal crystals (Structure 1) and “needle”‐shaped crystals (Structure 2) confirmed the COD structure for both (Table S1, Supporting Information). Binding of OEG_4_‐(IP5)_2_ to CaOx was further validated by inductively coupled plasma optical emission spectrometry analysis of ultrafiltered solution, showing the loss of detectable phosphorus signal from the compound at the presence of CaOx (Table S2, Supporting Information). Importantly, OEG_4_‐(IP5)_2_ addition drastically reduced CaOx bulk crystallization, resulting in very few detectable crystals at *t* = 7 h with 500 nm to 10 µm of compound (Figure S8B, Supporting Information).

Taken together, these results indicate that OEG_4_‐(IP5)_2_ alters the crystallization process at several stages, namely crystal nucleation and growth kinetics, as well as crystal polymorphism and shape (Figure 3C).

### 2.5 IP6 Analogues Block CaOx Adhesion to Renal Epithelial Cells In Vitro

**Figure 4:**
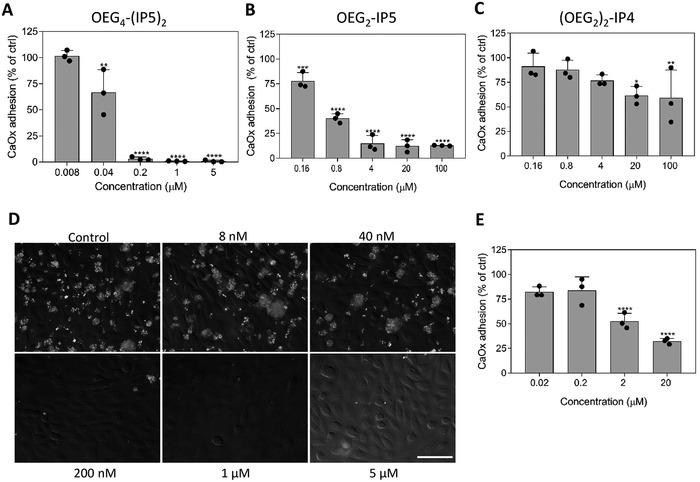
Inhibition of CaOx adhesion to renal epithelial cells by IP6 analogues. Anti‐adhesive effects of A) OEG_4_‐(IP5)_2_, B) OEG_2_‐IP5, and C) (OEG_2_)_2_‐IP4 on CaOx attachment to RPTEC/TERT1 cells in vitro were assessed by differential interference contrast microscopy followed by quantification of the crystal occupied area. Cells at confluence were treated with 150 µg cm^−2^ CaOx premixed with compound for 30 min, before washing, fixation, and imaging. D) Representative images of CaOx premixed with/without OEG_4_‐(IP5)_2_ adhering to the confluent cell layer (63× oil objective, scale bar: 50 µm). E) To assess the capacity of OEG_4_‐(IP5)_2_ to remove already bound CaOx of cell layers an adapted version of the assay described above was performed. RPTEC/TERT1 cells were incubated with CaOx for 30 min, unbound CaOx was removed by washing with PBS and cells were further incubated with OEG_4_‐(IP5)_2_ or medium as a control for 2 h. Data was normalized to the control (medium without inhibitor) and presented as mean + SD (*N* = 3, one‐way ANOVA with Dunnett's multiple comparison ****p < 0.0001, ***p < 0.001, **p < 0.01, *p < 0.05 compared to ctrl).

It was previously reported that the attachment of CaOx crystals to the renal epithelium plays a critical role in triggering further crystal aggregation and growth, leading to tubular obstruction, kidney stone formation, and renal injury.^[^ 7, 8, 9 ^]^ Therefore, anti‐adhesive effects of chosen IP6 analogues on CaOx crystals to renal proximal tubular epithelial cells (RPTECs) in vitro were investigated by differential interference contrast (DIC) microscopy. With this approach, a similar dependency of the amount of phosphate groups present on the molecule on their potency to inhibit CaOx adhesion was observed as in our CaOx growth inhibition assays. Comparison of OEG_4_‐(IP5)_2_, OEG_2_‐IP5, and (OEG_2_)_2_‐IP4 exemplifies this relationship, with concentrations of 200 nm, 4 µm, and above 100 µm, respectively, leading to <25% of CaOx adhesion compared to the positive control (without inhibitor) (**Figure** **4**A–D). Citrate treatment did not lead to any detectable protection against CaOx adhesion up to 100 µm (Figure S9, Supporting Information). Additionally, OEG_4_‐(IP5)_2_ not only inhibited CaOx adhesion, but also reversed the binding of pre‐bound CaOx crystals at 2 µm (Figure 4E; Figure S10, Supporting Information).

### 2.6 CaOx‐Induced Cellular Transcriptomic Changes Are Prevented by OEG_4_‐(IP5)_2_ In Vitro

**Figure 5:**
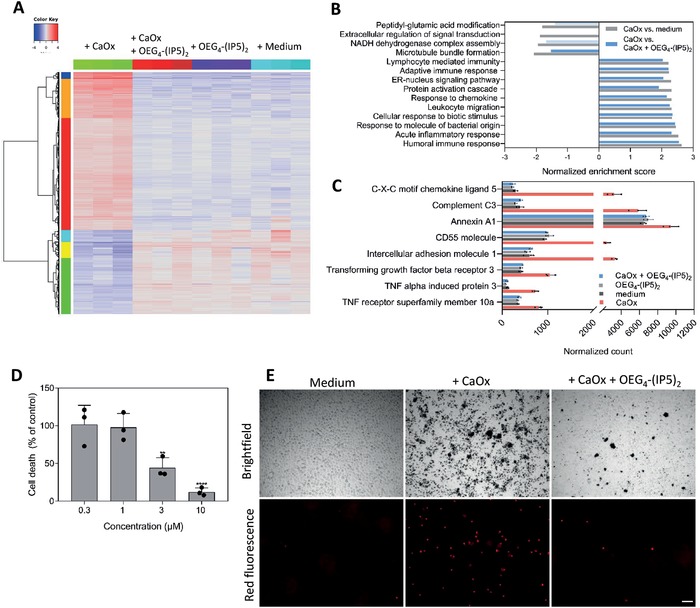
CaOx‐induced cellular injury of renal epithelial cells is prevented by OEG_4_‐(IP5)_2_ in vitro. A) Heatmap and hierarchical clustering of relative gene expression levels determined by RNA sequencing. Top 2000 differentially expressed genes between CaOx versus CaOx + OEG_4_‐(IP5)_2_ with *p* < 0.01, log_2_ratio = 0.5, and respective expression levels in the other treatment groups are plotted (red—relatively upregulated, blue—relatively downregulated). B) Gene set enrichment analysis of gene expression levels in the CaOx versus medium group and corresponding values in the CaOx versus CaOx + OEG_4_‐(IP5)_2_ group. Top gene ontology terms and corresponding normalized enrichment score for differentially up‐ or downregulated genes in the CaOx versus medium control group are plotted (top 10 upregulated gene ontology terms, all FDR ≤ 0.05 and downregulated gene ontology terms with FDR ≤ 0.05 are shown). Corresponding values for differentially expressed genes in the CaOx versus CaOx + OEG_4_‐(IP5)_2_ group are given (FDR ≤ 0.05 is indicated by dark blue, FDR > 0.05 by light blue). C) Normalized count of gene transcripts involved in inflammatory and immune response, cellular signaling, and extracellular matrix production in the different treatment groups (mean + SD, *N* = 3). D) Dose‐dependent decrease in the number of dead cells by pre‐incubation of CaOx with OEG_4_‐(IP5)_2_ before treatment of RPTEC cells in comparison to CaOx‐only treated cells was assessed by a viability stain (mean + SD normalized to CaOx ctrl, one‐way ANOVA with Dunnett's multiple comparison, ****p < 0.0001, **p < 0.01). E) Representative brightfield images showing CaOx deposition (black spots) and red fluorescence images indicating cell death of cells receiving no treatment (medium), CaOx only, or CaOx + 10 µm OEG_4_‐(IP5)_2_ treatment (10× objective, scale bar: 100 µm). (*N* = 3 for all experiments.)

Upon cell interaction, CaOx crystals have been suggested to elicit cytotoxicity, inflammation, and fibrosis.^[^ 11, 12, 13 ^]^ Due to the anti‐adhesive effects of OEG_4_‐(IP5)_2_, we hypothesized that the compound could further prevent downstream effects of CaOx crystals on tubular cells. RNA sequencing (RNAseq) of RPTECs showed a similar gene expression profile between medium control, OEG_4_‐(IP5)_2_ control, and cells treated with CaOx pre‐mixed with OEG_4_‐(IP5)_2_ (CaOx + OEG_4_‐(IP5)_2_), while CaOx crystals alone induced drastic changes (**Figure** **5**A). Gene set enrichment analysis of differently expressed genes in the CaOx versus medium samples revealed CaOx‐induced alterations of gene expression mainly involved in immune and inflammatory responses, which coincides with literature,^[^ 11, 12, 13 ^]^ and in structural changes (e.g., microtubule organization) as well as protein modification and signaling effects (e.g., peptidyl‐glutamic acid modification, ER‐nucleus signaling pathway) (Figure 5B). Further, effects on NADH dehydrogenase complex assembly and extracellular regulation of signal transduction were detected. However, those effects might have been caused by increased mitochondrial gene expression in one medium control sample, which cannot be explained. Comparing the top 10 enriched gene sets of differentially expressed genes in CaOx versus medium samples to CaOx versus CaOx + OEG_4_‐(IP5)_2_ samples showed similar pathway enrichment, indicating that premixing OEG_4_‐(IP5)_2_ with CaOx can prevent CaOx‐induced transcriptomic changes (Figure 5B). Small differences comparing CaOx versus medium to CaOx versus CaOx + OEG_4_‐(IP5)_2_ were observed in the gene sets “protein activation cascade” and “peptidyl‐glutamic acid modification”, which might represent small changes still occurring despite OEG_4_‐(IP5)_2_ treatment. This is further supported by comparison of CaOx + OEG_4_‐(IP5)_2_ versus medium sample groups. While no differentially expressed genes with an FDR ≤ 0.05 and a log_2_ratio ≥ 0.5 were found between those groups, gene set enrichment analysis similarly revealed enrichment in protein activation cascade and protein localization (Figure S11, Supporting Information). Thus, while major gene expression changes are largely inhibited by OEG_4_‐(IP5)_2_, slight alterations might still be present.

### 2.7 OEG_4_‐(IP5)_2_ Inhibits CaOx‐Induced Changes in Signaling Pathway and Cell Surface Genes

Next, we compared expression levels of selected genes that are either reported in literature to be involved in a cellular response to CaOx and/or were found to be among the top deregulated genes in this study. TNF receptor and TGF beta signaling pathways are assumed to play a role in the induction of an immune response in CaOx nephropathies.^[^ 35, 36 ^]^ Indeed, we found elevated expression of TNF alpha and TGF beta signaling pathway genes, such as TNF receptor superfamily member 10a, TNF alpha induced protein 3, and TGF beta receptor 3, in the CaOx group compared to all other groups (Figure 5C). Further, cell surface glycoproteins, in particular annexin II, were reported to be upregulated in hyperoxaluric animal models and suggested to facilitate CaOx adhesion.^[^ 8, 35 ^]^ In accordance, we observed an upregulation of numerous cell surface glycoproteins, especially a striking increase in intercellular adhesion molecule 1 transcript levels, but also elevated expression levels of CD55 and Annexin A1 with CaOx addition in comparison to medium, CaOx + OEG_4_‐(IP5)_2_, or OEG_4_‐(IP5)_2_ only groups. Additionally, several complement proteins and C‐X‐C motif chemokine ligands, for example, complement C3 and C‐X‐C chemokine ligand 5, were among the top upregulated genes, which were not reported previously to be involved in the cellular response to CaOx (Figure 5C).

CaOx‐induced upregulation of immune and inflammatory pathways might result in the reported cytotoxicity of CaOx crystals on renal epithelial cells.^[^ 11 ^]^ Indeed, exposing cells to CaOx crystals triggered plasma membrane damage, as evidenced by ethidium homodimer‐1 staining, which was prevented by OEG_4_‐(IP5)_2_ in a dose‐dependent manner (Figure 5D,E). Brightfield imaging further supported the reduced adhesion of CaOx to RPTECs with 10 µm OEG_4_‐(IP5)_2_ after washing (Figure 5E).

To summarize, these results suggest that by reducing cell–crystal interactions OEG_4_‐(IP5)_2_ treatment can largely prevent CaOx‐induced downstream responses, such as inflammatory signaling pathways.

### 2.8 OEG_4_‐(IP5)_2_ Reduces Renal CaOx Deposition and Kidney Injury In Vivo

**Figure 6:**
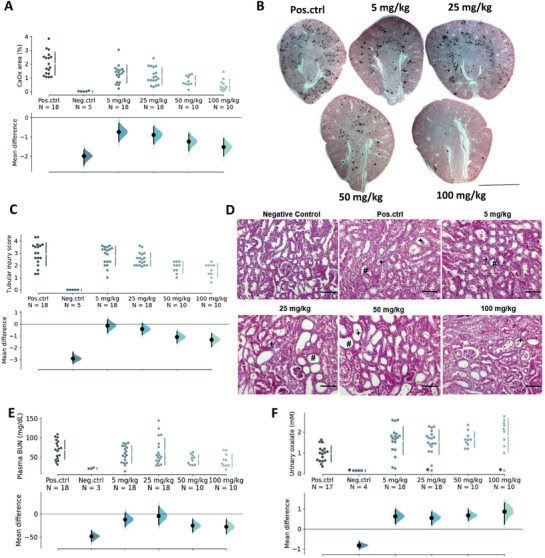
OEG_4_‐(IP5)_2_ reduces renal CaOx deposition and injury in a mouse model of renal CaOx crystallization. C57B16/N mice were fed an oxalate‐enriched and calcium‐depleted diet to induce renal CaOx crystallization and concurrently treated with indicated concentrations of OEG_4_‐(IP5)_2_ s.c. twice daily for seven consecutive days. A) Kidney sections of mice were stained for CaOx deposition by Pizzolato staining and dark areas indicating CaOx were quantified. B) Representative images of Pizzolato stained kidney sections are shown (scale bar: 2 mm). C) Kidney sections stained with periodic acid–Schiff stain were scored for kidney injury. D) Representative images of stained kidney sections are shown and areas of tubular injury highlighted (*—necrotic areas; #—dilated tubules; +—tubular casts; scale bar: 200 µm). E) Plasma blood urea nitrogen (BUN) was measured at day 7. F) Urinary oxalate levels were measured with an oxalate kit (LTA S.r.l.) at day 7. *—urinary oxalate measurements yielding values less than the lower threshold of 0.1447 mm were set to 0.1447 mm. “Pos.ctrl” indicates mice receiving the oxalate‐enriched and calcium‐depleted diet, and vehicle injections; “Neg.ctrl” mice represent healthy mice on a normal diet and receiving no treatment. The mean difference for five comparisons against the shared control “Pos.ctrl” is shown in the above Cumming estimation plot. The raw data is plotted on the upper axes with the line break denoting the mean value of each group and the lines indicating the standard deviation. On the lower axes, mean differences are plotted as bootstrap sampling distributions. Each mean difference is depicted as a dot. Each 95% confidence interval is indicated by the ends of the vertical error bars.

Due to the promising in vitro results of OEG_4_‐(IP5)_2_, an in vivo proof‐of concept study in a mouse model of CaOx‐induced nephrocalcinosis was performed. Crystallization was induced by an oxalate‐enriched and calcium‐depleted diet, as previously reported.^[^ 37 ^]^ Concurrently, mice were treated with OEG_4_‐(IP5)_2_ subcutaneously twice daily at the indicated doses for 7 days before sacrifice. Kidney sections stained by Pizzolato staining to identify CaOx deposition revealed a dose‐dependent reduction in renal CaOx with OEG_4_‐(IP5)_2_, which was significant for all treatment groups compared to the vehicle control group (**Figure** **6**A,B; Table S3, Supporting Information). Plasma blood urea nitrogen suggested improved excretory kidney function with OEG_4_‐(IP5)_2_ treatment at 100 mg kg^−1^ OEG_4_‐(IP5)_2_ treatment compared to vehicle control (Figure 6E; Table S3, Supporting Information). Kidney injury was reduced in a dose‐dependent manner as evidenced by periodic acid–Schiff staining of kidney sections and scoring of tubular injury (Figure 6C,D; Table S3, Supporting Information). As expected, urinary oxalate was drastically increased at day 7 of oxalate‐enriched diet compared to healthy mice. Interestingly, urinary oxalate levels were further elevated by all doses of OEG_4_‐(IP5)_2_ compared to vehicle control on day 7 (Figure 6F), suggesting a greater efflux of oxalate that was not retained within the kidneys consistent with the lower CaOx crystal deposition within the kidneys (Figure 6A). Additionally, a higher amount of CaOx crystals in the collected urine was detected in OEG_4_‐(IP5)_2_‐treated animals compared to vehicle control (Figure S12, Supporting Information). These data might suggest an increased urinary excretion of oxalate and/or small CaOx crystals due to a delay in crystallization and growth, as well as shielding of CaOx–cell interactions. No significant changes of animal weight or other signs of compound toxicity were observed (Figure S13, Supporting Information).

Together, these results support in vivo efficacy of OEG_4_‐(IP5)_2_ to reduce renal CaOx crystallization and deposition in the kidneys. The study further confirms kidney‐protective effects of OEG_4_‐(IP5)_2_, presumably due to reduced CaOx crystallization and tissue interactions mediated by the compound.

## 3 Discussion

CaOx crystal induced nephropathies comprise a diverse set of kidney disorders with limited treatment options.^[^ 1, 2, 3, 4, 5 ^]^ While crystallization inhibitors have been suggested as a possible treatment modality decades ago, investigations so far have not resulted in the clinical development of a promising CaOx crystallization inhibitor that could be used for the treatment of CaOx nephropathies. In this study, we report the identification and characterization of a potent CaOx crystallization inhibitor.

Leveraging our expertise in IP6 engineering^[^ 29, 30, 38, 39 ^]^ and the implementation of an image‐based screening approach enabled us to rapidly assess the effect of different modifications on the dynamics of CaOx crystallization under simulated pathophysiological conditions. The activity of IP6 analogues was highly dependent on the number of negative charges of the molecule, presumably being the driving force of binding between Ca^2+^ ions on the crystal surface and the molecules, thereby leading to inhibition of crystal growth.^[^ 26, 40 ^]^ The relationship between negative charge and inhibitory efficacy did not appear to be linear, with a drastic jump between mono and divalent IP5 (OEG_2_‐IP5 and OEG_4_‐(IP5)_2_), and leveling off towards trivalent IP5 ((OEG_4_)_3_‐(IP5)_3_), indicating the importance of geometric effects of phosphate group distribution on the activity of the molecules.^[^ 25 ^]^

Comparing in vitro efficacy of CaOx crystallization inhibitors across different studies is challenging, due to the variation in testing conditions and methods used. However, two characteristics of the present study make us confident to have discovered an exceedingly efficient CaOx crystallization inhibitor. First, most previous studies focus on the inhibition and face‐specific alteration of COM growth,^[^ 20, 21, 22, 25 ^]^ while we demonstrate complete inhibition of both COM and COD crystallization over hours at low inhibitor concentrations. Stabilization and coating of early‐stage nanoparticles by OEG_4_‐(IP5)_2_, paired with the delay and reduction in bulk crystallization should reduce tubular crystallization and retention of CaOx, considering that the passing time of renal tubular fluid ranges from 3–4 min.^[^ 17 ^]^ Upon crystallization, our lead compound shifts COM towards COD crystallization, which might be of therapeutic benefit due to the observed lower affinity of the latter to epithelial cell membrane molecules, and thus lower intratubular retention.^[^ 41 ^]^

Second, except for poly(acrylic acid),^[^ 42 ^]^ no data on in vivo efficacy of tested CaOx inhibitors is available, despite great efforts to optimize the structure of, for example, peptidic inhibitors or citrate derivatives.^[^ 21, 22 ^]^ Herein, we show reduced renal deposition of CaOx by subcutaneous administration of OEG_4_‐(IP5)_2_ in an acute model of nephrocalcinosis. An increase in urinary oxalate and urinary CaOx crystals in the compound‐treated groups further suggests a decrease and/or delay of intratubular CaOx crystallization. Together, our comprehensive analysis provides compelling evidence of the divalent IP5 molecules' efficacy, which translated to effective prevention of CaOx deposition and kidney injury in vivo. The presented screening assay might serve as a useful framework to compare novel inhibitors in a rapid and affordable manner, allowing for a more efficient screening process and better means of comparison between studies.

Another, often neglected aspect of the impact of crystallization inhibitors in CaOx nephropathies is the effect of inhibitory molecules on the interplay between crystals and the renal epithelium, and the resulting kidney injury. Our in vitro studies revealed a radical decrease in CaOx crystal adhesion by OEG_4_‐(IP5)_2_, possibly due to competitive binding between negatively charged extracellular matrix or cell surface molecules and OEG_4_‐(IP5)_2_ to Ca^2+^ on the crystal surface.^[^ 43 ^]^ Shielding of CaOx–cell interactions might provide an additional pathway to reduce CaOx deposition in the kidneys by increasing the efflux of already formed crystals, which is evidenced by an increase in urinary crystals of OEG_4_‐(IP5)_2_‐treated mice versus the vehicle control group. Blockage of crystal–cell interactions likely prevented cellular downstream responses related to immune and inflammatory responses and structural changes as shown in vitro by RNAseq. A striking upregulation of complement cascade genes, C‐X‐C motif chemokine ligands, and cell surface glycoproteins was induced by CaOx treatment of RPTECs. By inhibiting cellular adhesion, OEG_4_‐(IP5)_2_ almost completely prevented these changes in gene expression levels. Minor alterations in the cellular transcriptome were still detected, and albeit marginal, these might indicate some effects of the presence of unbound CaOx or elevated free oxalate and calcium levels. Cyto‐protective effects of OEG_4_‐(IP5)_2_ were further confirmed in vivo by decreased plasma blood urea nitrogen levels and a decreased tubular injury score. However, also in this setting the values did not revert to negative control levels. Thus, over prolonged exposure to small CaOx crystals or elevated oxalate and calcium concentrations, kidney injury might still occur. The results warrant further in‐depth investigation of CaOx–cell interactions and modulation thereof with OEG_4_‐(IP5)_2_.

While in this study we unravel an exciting new class of CaOx inhibitors with exceptional efficacy, potential toxicity of the compound, and in vivo effects on the crystallization process, in particular in ureter and bladder, need to be further assessed in pre‐clinical studies. So far only a pilot animal study was carried out, aiming at confirming inhibitory effects on renal CaOx deposition and CaOx‐induced renal injury. To fully grasp the wide clinical spectra of CaOx nephropathies other animal studies using different models, that is, resembling chronic versus acute disease states, should be conducted.

To conclude, in this work we describe the development of a powerful class of CaOx inhibitors with the lead compound, OEG_4_‐(IP5)_2_, acting on two steps common to CaOx nephropathies, namely the direct inhibition of the crystallization process and the interference with the kidney‐damaging CaOx–cell interactions. Together, these effects likely favor efflux of oxalate or CaOx precursor particles shielded by OEG_4_‐(IP5)_2_, preventing CaOx‐induced kidney injury. The nanomolar efficacy is, to the best of our knowledge, unparalleled in inhibiting CaOx crystallization and therefore, the new CaOx inhibitors hold promise as a novel therapeutic modality in CaOx‐related nephropathies.

## 4 Experimental Section

##### Materials

IP6 analogues were custom synthesized by Chimete Srl (Tortona, Italy). Mass and ^1^H‐NMR spectra were taken by the provider to confirm the structure and the compounds were used as provided. Phytic acid dodecasodium salt was purchased from Biosynth AG (Thal, Switzerland). Pooled human urine was purchased from Lee Biosolutions (Maryland Heights, MO, USA). Oxalate Assay kit (MAK315), Calcium Colorimetric Assay kit (MAK022), Bis‐Tris, sodium oxalate (NaOx), formalin solution neutral buffered 10%, periodic acid–Schiff kit, and Mowiol 4‐88 were purchased from Sigma‐Aldrich (St.Louis, MO, USA). Sodium chloride (NaCl) and calcium chloride (CaCl_2_) dihydrate were obtained from Merck (Kenilworth, NJ, USA). Calcium oxalate (CaOx) monohydrate was purchased from abcr (Karlsruhe, Germany). 8‐well glass bottom slides (80 827) were purchased from ibidi (Martinsried, Germany). Trisodium citrate dihydrate analytical grade, Nunc Lab‐Tek II chamber slides, standard cell culture plates and reagents, and the LIVE/DEAD Viability/Cytotoxicity kit for mammalian cells were purchased from Thermo Fisher Scientific (Rochester, NY, USA). RPTEC/TERT1 cells, ProxUp basal medium, and supplements were obtained from Evercyte (Vienna, Austria). RNeasy kit was purchased from Quiagen (Hilden, Germany) and TrueSeq RNA kit from Illumina (San Diego, CA, USA). Male C57BL/6N mice were purchased from Charles River Laboratories (Sulzfeld, Germany). Plasma BUN kit was obtained from DiaSys GmBH (Holzheim, Germany). Oxalate kit used for the in vivo samples was purchased from LTA S.r.l. (Vernio, Italy). 10 kD spin column (ab93349) were purchased from Abcam (Cambridge, UK).

##### CaOx Screening Assay and Analysis

Human urine was stored at −20 °C in 50 mL aliquots. After thawing, aliquots were centrifuged, filtered using a 0.45‐µm syringe filter, and pH adjusted to 6.2. Oxalate concentrations of human urine samples were determined using the Oxalate Assay kit following manufacturer's instructions, and found to be <100 µm for the samples used. Dilutions of sodium oxalate and compounds were prepared in Bis‐Tris buffer (50 mm Bis‐Tris, 150 mm NaCl, pH 6.2). Urine was mixed with compound in Eppendorf tubes, and subsequently sodium oxalate was added. The final assay mixture of 1 mL total volume contained 90% (v/v) urine, 5% (v/v) sodium oxalate (1 mm final concentration), and 5% (v/v) compound dilution. Immediately upon sodium oxalate addition, the final assay mixture was vortexed and added to the imaging slide (8‐well glass bottom chamber, ibidi). 400 µL per well were added and for each sample two wells were prepared. Crystallization was assessed after a 7‐h incubation at room temperature (RT), using a Leica DM 6000B microscope (Leica Microsystems, Wetzlar, Germany) in brightfield mode. For visualization and quantification of CaOx crystals, images were taken with an HCX PL FLUOTAR 40×/0.75 dry and an HC PL FLUOTAR L 20×/0.4 dry objective, respectively. Images were obtained in TL‐BF mode, using the ANT filter cube. For quantification, two wells per sample with five images in each well were used per experiment. Without addition of sodium oxalate no crystallization was observed.

For the characterization of CaOx crystallization over time, the same assay was performed without addition of inhibitor, and images were taken after 1, 2, 4, 7, and 24 h at RT. Inhibition of seeded crystals was evaluated in a similar manner. Crystallization was induced with 1 mm sodium oxalate in urine as described above, followed by compound addition to each well after a 1.5 h incubation at RT. Samples were imaged before compound addition at *t* = 1.5 h and after 7 h total incubation time.

Quantification and classification of crystals was performed in Matlab (MathWorks, Natick, MA, USA, version R2016b or R2018b). In brief, crystals were segmented using edge detection and watershed algorithms, and then shape (area, perimeter, major axis and minor axis length, eccentricity, and circularity), intensity (variance, mean, minimum and maximum intensity, intensity distribution), and texture (correlation, contrast, homogeneity, and energy) features for each crystal were extracted. The extracted features served as input for a semi‐supervised classification approach to distinguish COM (class 0), COD (class 1), not defined structures (class 2, n.d.), and background noise (class 3). Training data was obtained by annotating crystals of single images using single features. This approach was possible due to the fact that in single images, different types of crystals can be easily distinguished based on a single feature, such as crystal size or maximum signal intensity. However, across different images single features were not sufficient anymore to provide accurate classification due to changes in, for example, size or intensities under different sample conditions applied. With the annotated training data different training algorithms were compared using the Classification Learner App. A cubic SVM classifier gave the best results and was used for classification of testing images.

Annotated crystals of thirteen training images of different experiments and conditions were used for classifier training, resulting in a total of 1710 annotated crystals, with the highest number of crystals representing class 0 and 1 (COD and COM). Performance of the trained classifier was evaluated by a fivefold cross validation with an overall accuracy of 90.9%. Per class performance was assessed by calculating precision and recall, and the F1 score was used as a harmonic mean between these two. For additional confirmation of classifier performance, images not included in the training set were classified using the trained classifier and output overlaid on input images (Figure S1, Supporting Information). As expected, the two common classes with distinct shapes, COM and COD, show better performance due to distinct morphology and drastically higher number of training examples.

Difficulties with segmentation of touching crystals and errors in the labeling of training data were observed. Furthermore, overfitting of the classifier might be a problem, due to changes in light intensity typical for brightfield microscopy and focus plane. The quantified output was compared with the images to ensure correct output of the image analysis pipeline.

COM crystals, which appeared in high numbers were analyzed further for single crystal size distribution. Size distribution analysis for COD crystals was not measured, due to the low number of COD and thus the higher impact of misclassifications.

All testing images were processed using the Batch Processing App. Efficacy of compounds to inhibit CaOx crystallization was assessed by the total area per field of view for each crystal type. The sum of the total area of the three crystal classes—COM, COD, and n.d., was normalized to the control (without inhibitor) within each experiment (*N* = 3). Due to the inherent variability of the crystallization process itself, normalization within each experiment decreased the effect of inter‐experimental variability.

Analysis scripts were written in Matlab (version 2016b and 2018b) and are available on: https://github.com/kletziclimbs/CaOx-image-analysis. For size distribution analysis, the area measurements of single COM crystals were extracted and used as an input for a distribution plot (measurements of *N* = 3 experiments, each experiment with 2 wells per sample and 5 images per well). The following script on MathWorks file exchange was used: distributionPlot.m (https://ch.mathworks.com/matlabcentral/fileexchange/23661-violin-plots-for-plotting-multiple-distributions-distributionplot-m).

##### Scanning Electron Microscopy

CaOx crystals for SEM were prepared in Bis‐Tris buffer (50 mm Bis‐Tris, 150 mm NaCl, pH 6.2) containing final concentrations of 1 mm NaOx, 2 mm CaCl_2_, and the indicated concentration of compound. 12‐mm round glass coverslips were placed at the bottom of 24‐well plates. First, 20× stock solutions of CaCl_2_ and inhibitor, and 10× NaOx were prepared in Bis‐Tris buffer. Assay mixture was prepared in Eppendorf tubes by first adding 800 µL of Bis‐Tris buffer, followed by addition of 50 µL CaCl_2_ (20× concentration) and 50 µL of inhibitor (20× concentration) and vortexing. Then, 100 µL NaOx (10× final concentration) was added, the assay mixture was vortexed and immediately added to the prepared 24‐well plate. 400 µL per well and 2 wells per sample were prepared. Samples were incubated at RT for the indicated time. Representative images were taken using a Leica DM 6000B microscope (Leica Microsystems) in brightfield mode before samples were washed once with double distilled water and dried at RT. Samples were imaged by brightfield microscopy again after drying to confirm little effects of the drying process on crystal morphology. After drying, glass coverslips were mounted on SEM stubs with silver paint and coated with a 6‐nm layer of platinum/palladium using a CCU‐010 Metal Sputter Coater (Safematic, Bad Ragaz, Switzerland). Samples were imaged using a Magellan 400 FEI SEM microscope (ThermoFisher Scientific) in the secondary electron mode using the TLD detector.

##### Single Crystal X‐ray Diffraction

For the confirmation of crystal hydrate forms observed in the CaOx screening assay single crystal X‐ray diffraction (XRD) was performed on selected samples. COD crystals with a tetragonal bipyramidal shape were prepared as described in the CaOx screening assay with the addition of 300 nm OEG_4_‐(IP5)_2_. Stability of the crystal morphology was confirmed by light microscopy over 5 days, at which the sample was submitted for analysis (Structure 1, Table S1, Supporting Information). Crystal structure was analyzed with an XtaLAB Synergy, Dualflex, Pilatus 300K diffractometer (Rigaku, Tokyo, Japan). The crystal structure was deposited on the CCDC depository under the number 1960751. For the long needle‐shaped crystals, formed upon high OEG_4_‐(IP5)_2_ concentrations conditions of the CaOx screening assay needed to be slightly adapted to ensure large enough growth of the crystals for single crystal XRD analysis. Therefore, crystals were grown in a solution of Bis‐Tris buffer (50 mm, 150 mm NaCl, pH 6.2) containing 2.5 mm CaCl_2_, 1.2 mm NaOx, and 25 µm OEG_4_‐(IP5)_2_, upon which needle‐shaped crystals, as observed in the original CaOx screening assay in urine, started appearing. Importantly, the same dose‐dependent change in crystallization pattern was observed upon lowering OEG_4_‐(IP5)_2_ concentrations. Needle‐shaped crystals were grown over 5 days and followed by light microscopy before structure determination as described above (Structure 2, Table S2, Supporting Information). COM crystals as observed in the CaOx screening assay were not analyzed by single crystal XRD due to their small size.

##### Calcium Detection Assay Kit

Calcium chelating properties of OEG_4_‐(IP5)_2_ and citrate were tested using the Calcium Colorimetric Assay kit (Sigma, MAK022). In brief, compound and standard dilutions were prepared directly in urine. Free calcium in solution was measured according to the manufacturer's instructions.

##### Cell Experiments

RPTEC/TERT1 human proximal tubule cells (Evercyte) were cultured in ProxUp basal medium (Evercyte, DMEM/Ham's F12, Hepes buffer, and GlutaMAX) mixed with ProxUp supplements (Evercyte) at 37 °C and 5% CO_2_ according to the manufacturer's recommendations. Cells were regularly tested for mycoplasma infection.

Cell viability was assessed using the LIVE/DEAD Viability/Cytotoxicity kit (ThermoFisher Scientific). Cells were cultured in 24‐well plates and 48 h after seeding (upon reaching 100% confluency) treated with 150 µg cm^−2^ calcium oxalate monohydrate (abcr) in ProxUp basal media pre‐mixed with compound. After a 48‐h incubation cell viability was determined by staining with the LIVE/DEAD Cytotoxicity kit (ThermoFisher Scientific), according to the manufacturer's instructions. Images were obtained by epifluorescence microscopy using a Leica DM 6000B microscope (Leica Microsystems, 3 wells per sample, 3 images per well) with the HI PLAN 10×/0.25 dry objective. Red fluorescence images were obtained using the TXR filter cube in FLUO mode and transmission images were obtained using the ANT cube and TL‐DIC mode. The number of red fluorescence dots was quantified.

For quantification of CaOx adhesion to RPTEC cells, cells were cultured on 8‐well Lab Tek chamber slides (Nunc, ThermoFisher Scientific), and 48 h after seeding, upon reaching 100% confluency, treated with COM (abcr) pre‐mixed with compound in ProxUp basal medium (pH adjusted to 6.9) for 30 min at 37 °C, 5% CO_2_. Cells were washed twice with phosphate buffered saline (PBS, pH 7.4), fixed with 10% neutral buffered formalin for 15 min at RT, and rinsed with PBS, followed by a final rinse with ddH_2_O. The chamber of the slide was removed and cells mounted with Mowiol. Images were obtained with a Leica DM 6000B microscope (Leica Microsystems) in TL‐DIC mode using the ANT cube and a HL PL APO 63×/1.4 oil objective (3 wells per sample; 8 images per well). The crystal occupied area was quantified.

All cell experiments were carried out in three independent replicate experiments. Matlab code for image analysis of cell experiments is available on https://github.com/kletziclimbs/CaOx-Cell-experiments.

##### RNA Sequencing

Cells were cultured as described in the cell viability assay and treated with a medium control (ProxUp basal medium only), 150 µg cm^−2^ calcium oxalate monohydrate (abcr), 150 µg cm^−2^ calcium oxalate monohydrate (abcr) pre‐mixed with 10 µm OEG_4_‐(IP5)_2_, or 10 µm OEG_4_‐(IP5)_2_. Total RNA was extracted using the RNeasy kit (Quiagen) according to the manufacturer's instructions. Four wells per sample group were prepared and total RNA extracted of those 4 wells pooled. mRNA was purified and RNAseq library was prepared using the TrueSeq RNA kit (Illumina). Sequencing was performed on a Novaseq 6000 (Illumina). Reads were aligned to the human reference genome GRCh38.p10 using the STAR tool (https://github.com/alexdobin/STAR) and transcripts quantified using the Kallisto program.^[^ 44 ^]^ Ensembl release 91 was used for the gene model definitions. For the heatmap and hierarchical clustering of significantly different genes the log_2_fold changes in comparison to the mean of all samples was calculated and log_2_fold changes >4 were set to 4. The heatmap was plotted using R software. Gene set enrichment analysis was performed on Webgestalt.org (v2019).^[^ 45 ^]^ Input for gene set enrichment analysis were gene lists with the ensemble gene id and a ranking score (−log_10_ * *p*‐value *sign (log_2_ratio)) as a metric for differential expression between two treatment groups. Differential expressed genes were compared to the gene ontology—biological process functional database and as a reference set the human genome—protein coding was used. For comparison of expression levels of selected genes the normalized gene count was used. Three independent experiments were performed.

##### Animal Studies

7–8 week‐old male C57BL/6N mice were obtained from Charles River Laboratories (Sulzfeld, Germany). Animals were housed in filter‐top cages with a 12‐h dark–light cycle and unlimited access to food and water throughout the duration of the study. Mice were randomized to either treatment by block randomization. Oxalate nephropathy and hyperoxaluria was induced by feeding mice a calcium‐free and high‐oxalate diet as previously described.^[^ 37 ^]^ High‐oxalate diet was prepared by adding sodium oxalate (50 µmol g^−1^) to a calcium‐free diet (Ssniff, Soest, Germany). At the same starting time point as the high‐oxalate diet, OEG_4_‐(IP5)_2_ or vehicle was administered twice daily by subcutaneous injection for 7 days. Blood was drawn at day 7 under isoflurane anesthesia from the tail vein. Plasma was separated by centrifugation at 11 200 g for 5 min. Spot urine was collected on day 7 before sacrifice and immediately acidified with 0.1 m HCl. Urine crystals were separated by centrifugation at 7168 g for 5 min. Separated crystals were placed on a glass slide, covered with a cover slip and imaged by polarized microscopy (Leica Microsystems). Urine was stored at −20 °C until further analysis. Plasma blood urea nitrogen (DiaSys GmbH), and urine oxalate (LTA S.r.l.), were estimated by following the manufacturers' instructions. Urinary oxalate measurements that were below the lower detection threshold of the kit (0.1447 mm) were set to the lower threshold, and the respective datapoints were marked in the figure. All mice were sacrificed by cervical dislocation at the end of the study. For histology, kidney tissue samples (one part of each kidney per animal) were collected, and immediately stored in 4% formalin for paraffin embedding. Kidney sections of 2 µm were stained with periodic acid–Schiff reagent, and the tubular injury was scored by assessing the percentage of necrotic tubules and presence of tubular casts. CaOx crystal deposits were stained by Pizzolato staining and quantified using Image J software.^[^ 37 ^]^ All assessments were performed by an observer blinded to experimental conditions. Data used in this report represent combined data of two studies. In the first study only positive control (vehicle injection), 5 and 25 mg kg^−1^ OEG_4_‐(IP5)_2_ were tested (*N* = 8 animals per group). In the second study all treatment groups (i.e., positive control, as well as 5, 25, 50, and 100 mg kg^−1^ OEG_4_‐(IP5)_2_) were performed (*N* = 10 animals per group). Negative control values of animals receiving a calcium‐free diet without sodium oxalate (*N* = 3–5) were used from a representative previous study to minimize the use of animals according to ethical requirements. Where reported data points were less than the total number of animals per group the required sample volume was not met. No animals died during the study. All animal experiments were performed in accordance with the European protection laws of animal welfare, and with approval by the local government authorities of Oberbayern, Germany (reference number: 55.2‐1‐54‐2532‐189‐2015).

##### Compound–CaOx Binding Assay

For compound–CaOx binding studies, CaOx crystallization was induced in Bis‐Tris buffer (pH 6.2) containing 5 mm equimolar of CaCl_2_ and NaOx, and 10 µm of OEG_4_‐(IP5)_2_ in a total volume of 1 mL. Negative control presents the same sample without OEG_4_‐(IP5)_2_, positive control contains OEG_4_‐(IP5)_2_ only. Samples were vortexed and incubated for 1 h at room temperature before centrifugation and filtration using a 10 kD spin column at 10 000 × *g* for 5 min to separate particles from the solution. The filtrate was diluted 1:10 in ddH_2_O and analyzed by inductively coupled plasma–optical emission spectrometry analysis using an ICP‐OES Shimadzu (Kyoto, Japan). As a Calibration curve P/S/Si 0.05–20 mg L^−1^ was used.

##### Data Analysis

Statistical analysis and graphs were prepared using Prism (GraphPad, La Jolla, CA, USA), unless otherwise stated. Data were expressed as mean + S.D. Ordinary one‐way ANOVA testing followed by post‐hoc Dunnett's multiple comparison were used for comparison of treatment to control group. For animal experiments, estimation graphics were plotted using the DABEST Python package in Python 3.6.^[^ 46 ^]^ Normality of animal data was assessed by D'Agostino and Pearson testing and found to be normally distributed. Hence, ordinary one‐way ANOVA was used for statistical significance testing as described above. Image analysis was carried out using Matlab version R2018b and R2016b (MathWorks) unless otherwise stated. Matlab scripts used for image analysis can be found here: https://github.com/kletziclimbs/.

##### Data Availability

The main data supporting the results are provided in the figures and supporting information. RNA sequencing raw data is available on the EMBL Nucleotide Sequence Database (ENA) under the accession number PRJEB34728. Crystal structure was deposited on the CCDC depository under the number 1960751 Representative images for all experiments are provided, further raw images are available upon request.

##### Code Availability

Matlab code for image analysis of the CaOx screening assay is available on github (https://github.com/kletziclimbs/CaOx-image-analysis). Matlab code for cell experiments is available on github: https://github.com/kletziclimbs/CaOx-Cell-experiments. ImageJ script for the quantification of the Pizzolato staining is available upon request.

## Conflict of Interest

M.E.I., J.‐C.L., and A.K. are co‐inventors of patents licensed to/owned by Inositec Inc., and M.E.I. and J.‐C.L. are shareholders thereof.

## Author contributions

M.E.I., J.‐C.L., and A.K. designed the study and wrote the manuscript. M.E.I. and J.‐C.L. supervised the project. A.K. performed all in vitro experiments, wrote the image analysis code, and analyzed all data. H.‐J.A., M.M., and S.R.M. designed and performed the in vivo study and provided feedback on the manuscript. Z.L. helped with the design and interpretation of SEM and XRD experiments, provided overall feedback, and wrote the manuscript.

## Supporting information

Supporting Information
