# Supplementary material for: Inhibitors of Calcium Oxalate Crystallization for the Treatment of Oxalate Nephropathies
Source: Adv Sci (Weinh). 2020 Feb 27;7(8):1903337. doi: 10.1002/advs.201903337 (PMC7175250; doi:10.1002/advs.201903337)
Supplement: Supplementary file 1 — Supporting Information [file ADVS-7-1903337-s001.pdf]

Copyright WILEY-VCH Verlag GmbH & Co. KGaA, 69469 Weinheim, Germany, 2020.

ADVANCED SCIENCE
Open Access

Supporting Information

for *Adv. Sci.,* DOI: 10.1002/advs.201903337

Inhibitors of Calcium Oxalate Crystallization for the Treatment of Oxalate Nephropathies

Anna Kletzmayr, Shrikant R. Mulay, Manga Motrapu, Zhi Luo, Hans-Joachim Anders, Mattias E. Ivarsson, and Jean-Christophe Leroux**

Copyright WILEY-VCH Verlag GmbH & Co. KGaA, 69469 Weinheim, Germany, 2018.

Supporting Information

Inhibitors of Calcium Oxalate Crystallization for the Treatment of Oxalate Nephropathies

Anna Kletzmayr, Shrikant R. Mulay, Manga Motrapu, Zhi Luo, Hans-Joachim Anders, Mattias E. Ivarsson, Jean-Christophe Leroux**

A

True class	Predicted class 0	1	2	3	Precision	Recall	F1-score
**0**	367	33	8	0	0.94	0.90	0.92
**1**	18	971	18	16	0.92	0.95	0.93
**2**	7	32	171	1	0.86	0.81	0.84
**3**	0	22	1	45	0.73	0.66	0.69

B

Classified image

Input image

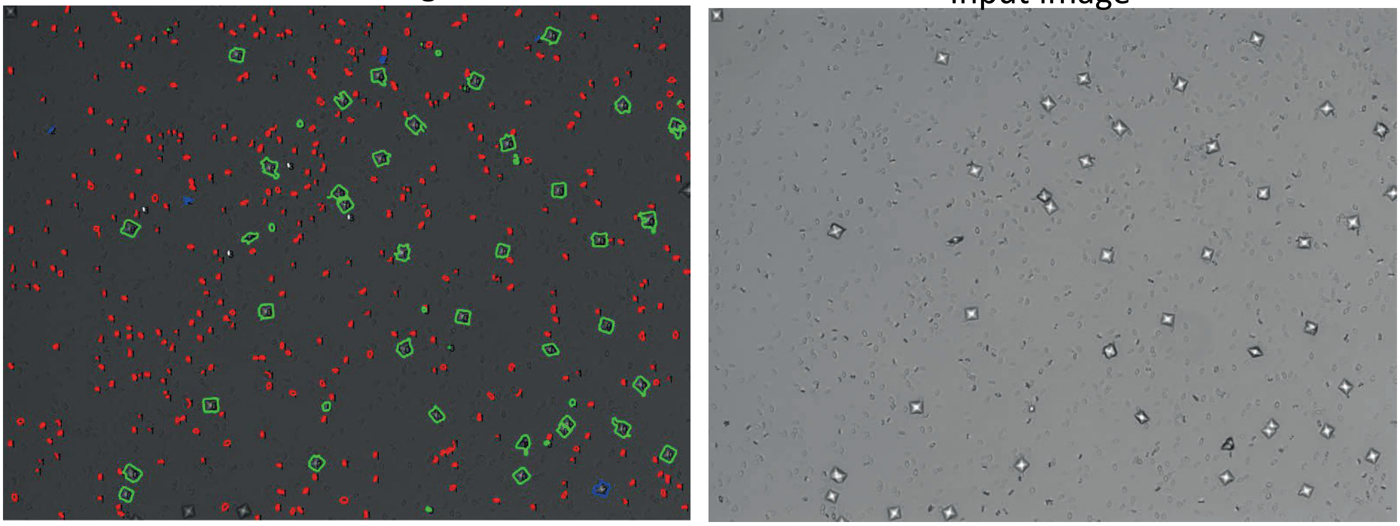

SFig.1: Evaluation of the image analysis and classification protocol for the CaOx growth screening assay. (A) Confusion matrix of the SVM classifier trained (class 0 – CaOx dihydrate (COD), class 1 – CaOx monohydrate (COM), class 2 – not defined (n.d)., class 3 – noise). Classifier was trained based on the input features of single segmented crystals from 13 pre-annotated training images (from different experiments and different conditions) and validated using a five-fold cross validation setup with an overall accuracy of 90.9%. Per-class performance was assessed by calculating precision, recall and F1-score. (B) Example of the results of the segmentation protocol and classifier output (left) on a representative image (right) not included in the training set (green: COD, red: COM, blue: n.d., white: noise).

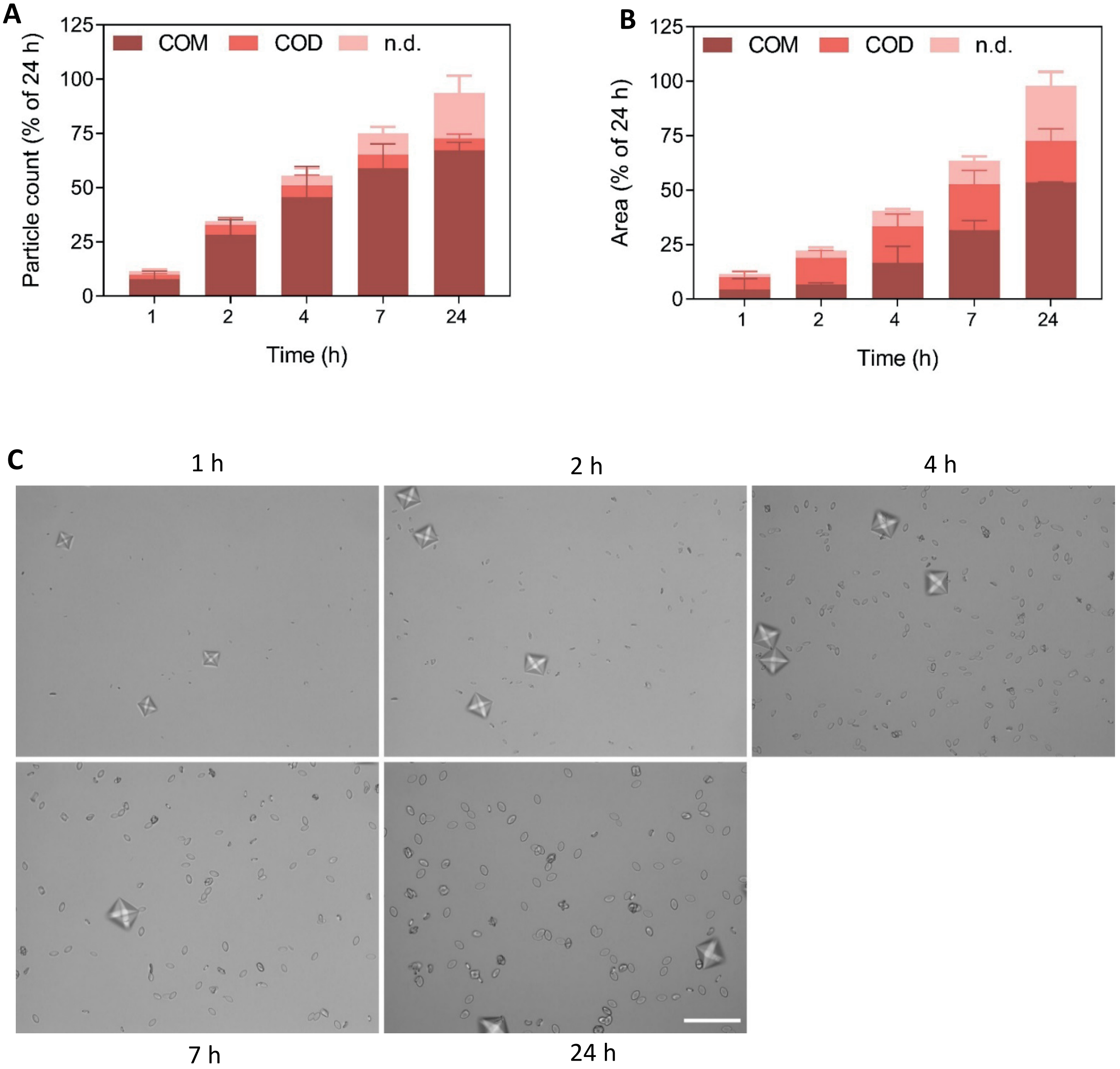

SFig.2: Characterization of the CaOx growth screening assay. CaOx crystallization in human urine was induced by spiking with 1 mM NaOx and followed over 24 h. Crystal number, area and hydrate forms were assessed by light microscopy followed by an automated image analysis. (A) Average total particle number per field of view for each crystal type normalized to the sample at t = 24 h is plotted. (B) Total surface area of the different hydrate forms normalized to the total surface area of the sample at t = 24 h is plotted (N = 3, mean + SD). (C) Representative images of the respective timepoints are shown (40x objective, scale bar: 50 µm).

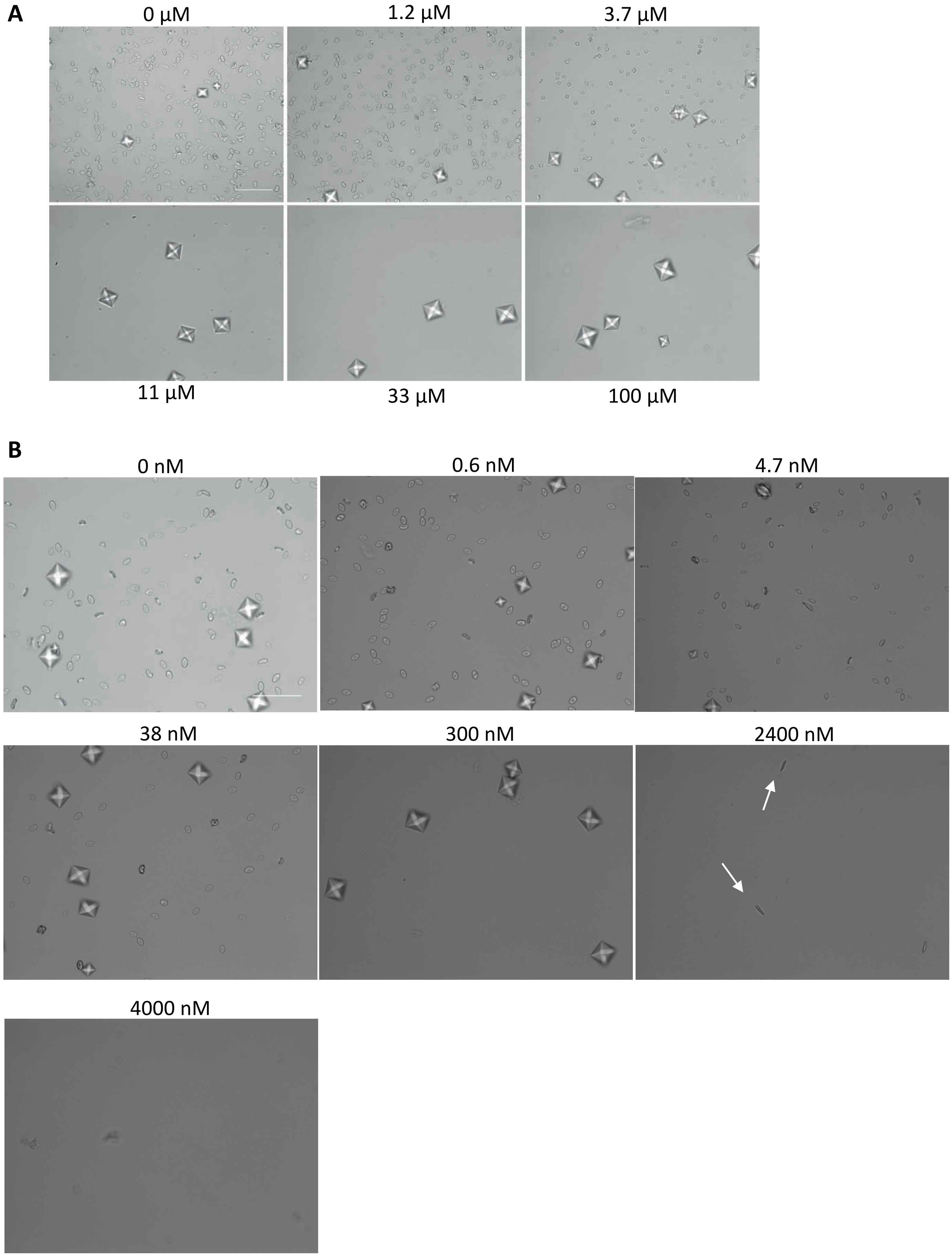

SFig.3: Results of the CaOx screening assay. Representative images of (A) OEG_2-IP5 and (B) OEG_4-$(IP5)_2$ showing the effect of increasing concentrations of the respective inhibitor on CaOx crystallization in human urine spiked with 1 mM NaOx at t = 7 h (scale bar: 50 µm, 40x objective). Needle-shaped COD crystals formed upon high OEG_4-$(IP5)_2$ concentrations are indicated by white arrows.

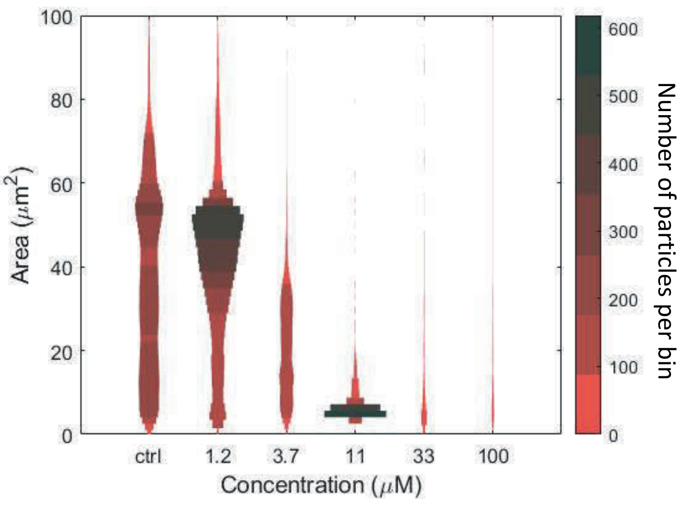

SFig.4: Effects of OEG$_2$-IP5 on COM single crystal size distribution. Images of the CaOx growth screening assay previously described were analysed for the effect of OEG$_2$-IP5 on particle size distribution of single COM crystals. Violin plot of single COM area is shown. Plot width indicates the number of COM per bin and the colorbar specifying absolute values (cumulative results of N = 3 independent experiments, ctrl – without inhibitor).

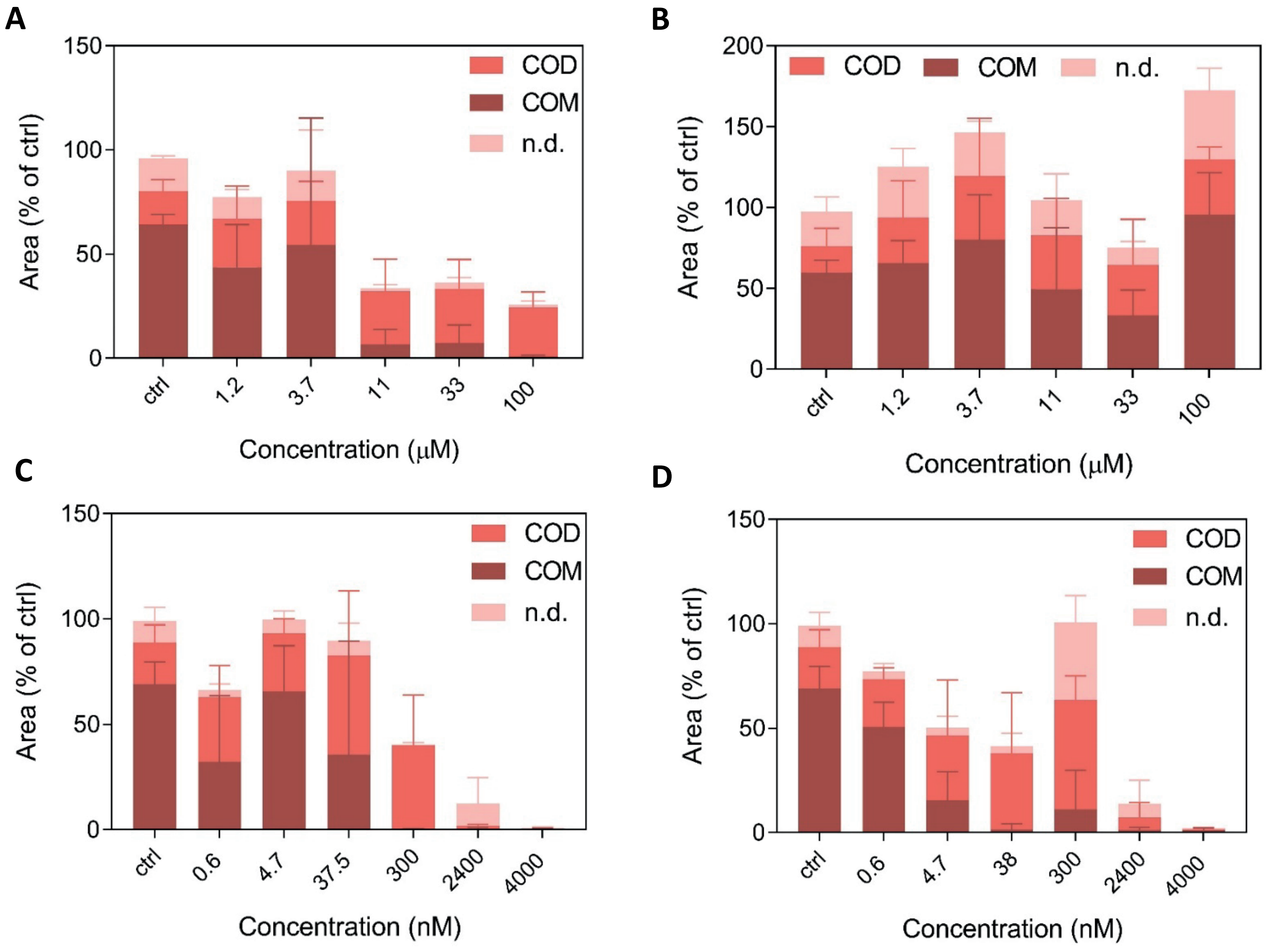

SFig.5: Results of the library screening - efficacy of additional compounds to inhibit CaOx bulk crystallization. Effects of (A) OEG$_{11}$-IP5, (B) citrate, (C) OEG$_{2}$-(IP5)$_{2}$ and (D) OEG$_{8}$-(IP5)$_{2}$ on CaOx bulk crystallization in human urine spiked with 1 mM NaOx were assessed by light microscopy at t = 7 h. The mean total area / field of view + SD for the respective crystal type normalized to the control (without inhibitor) is plotted (N = 3, COM – CaOx monohydrate, COD – CaOx dihydrate, n.d. – not defined).

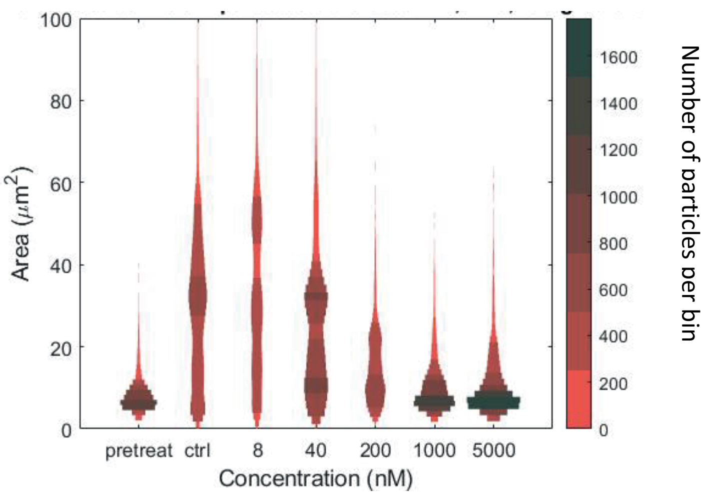

SFig. 6: COM stabilization by OEG_4-$(IP5)_2$. A modified version of the CaOx screening assay was performed, wherein inhibitor was added after CaOx seed formation at t = 1.5 h. Particle size distribution analysis of COM crystals indicates dose-dependent stabilization of COM crystals with the addition of OEG_4-$(IP5)_2$. Violin plot of single COM area is shown. Plot width indicates the number of COM per bin and the colorbar specifying absolute values. Pre-treat presents the sample at t = 1.5 h before addition of inhibitor, ctrl presents sample at t = 7 h without inhibitor, all other groups present the sample with added amount of inhibitor at the indicated concentration at t =7 h (cumulative results of N = 3 independent experiments).

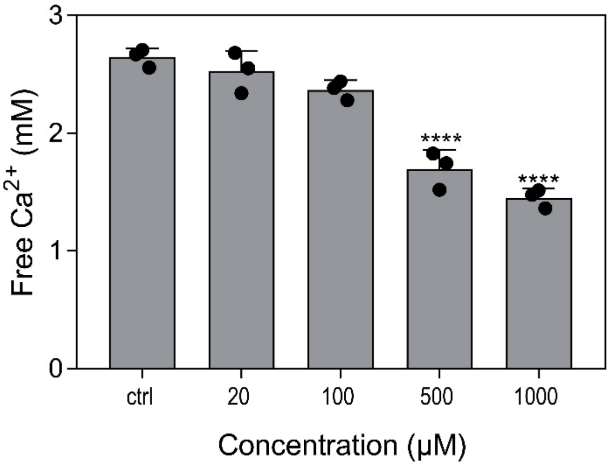

SFig.7: Calcium chelating properties of OEG_4-(IP5)$_2$. Prospensity of OEG_4-(IP5)$_2$ to reduce free calcium in human urine (pH 6.2) was measured using a colorimetric assay (N = 3, mean + SD, one-way ANOVA with Dunnett's multiple comparison, **** $p < 0.0001$ compared to control (without inhibitor)).

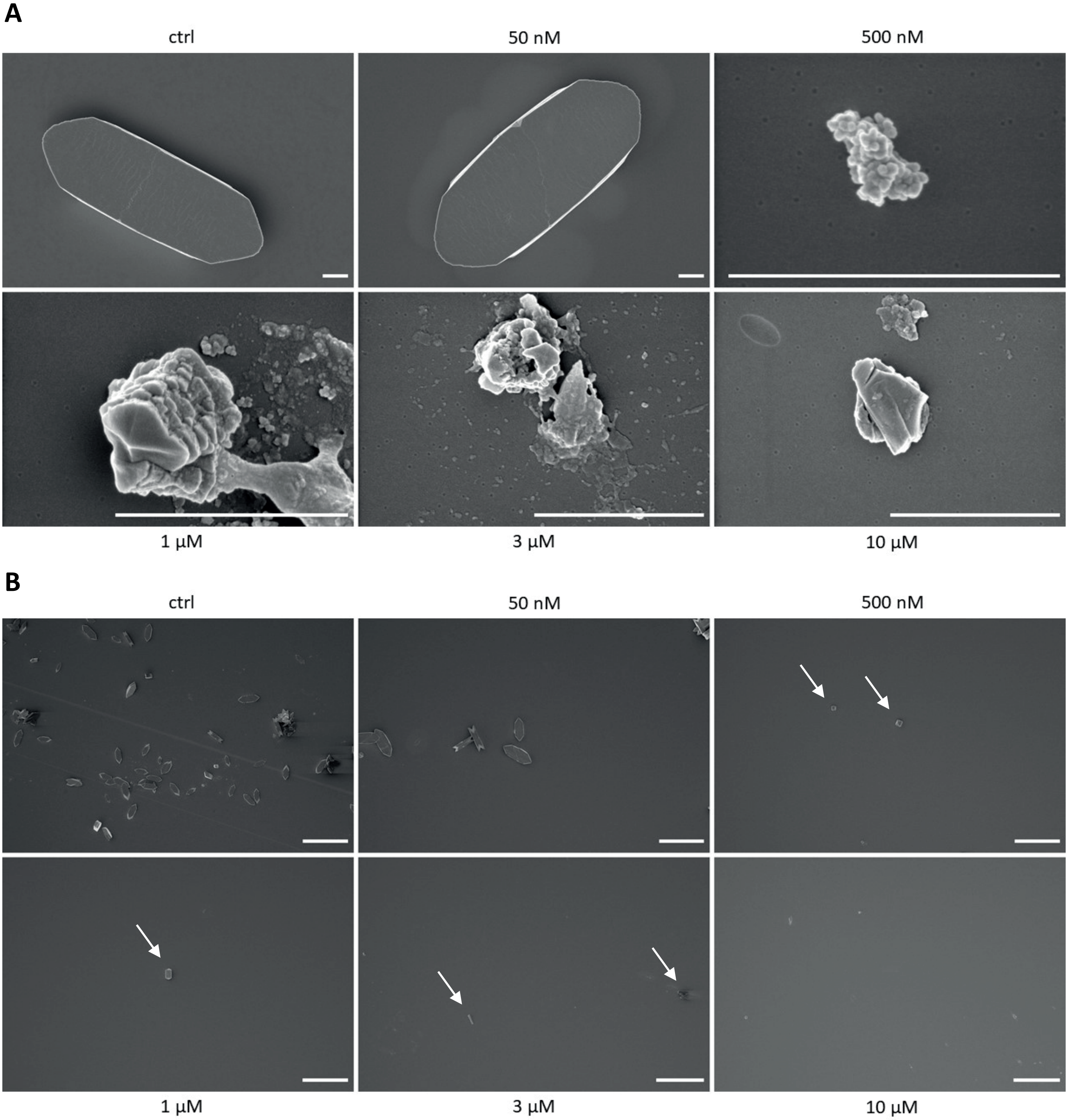

SFig.8: Dose-dependent inhibition of CaOx crystallization by OEG_4-$(IP5)_2$. (A) Representative SEM images of CaOx crystal growth in the presence of increasing doses of OEG_4-$(IP5)_2$ at t = 1 h (scale bar: 1 µm). (B) Representative overview SEM images of dose-dependent inhibition of CaOx crystallization with increasing doses of OEG_4-$(IP5)_2$ at t = 7h. Arrows indicate small CaOx crystals (scale bar: 20 µm, control – without inhibitor).

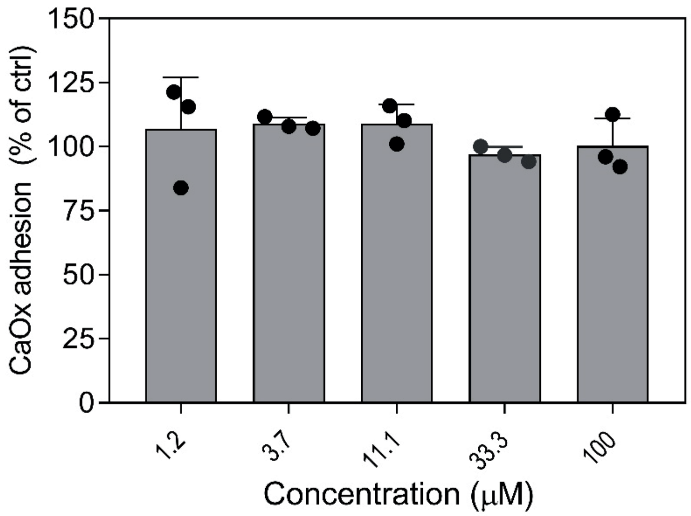

SFig.9: Inhibition of CaOx adhesion to renal epithelial cells by citrate. Anti-adhesive effects of citrate on CaOx attachment to RPTEC/TERT1 cells *in vitro* were assessed by light microscopy followed by quantification of the crystal occupied area. Cells at confluence were treated with 150 μg/cm^2 CaOx premixed with citrate for 30 min, before washing, fixation and imaging. (N = 3, mean + SD normalized to the CaOx ctrl (without inhibitor), one-way ANOVA with Dunnett's multiple comparison, **** $p < 0.0001$, *** $p < 0.001$, ** $p < 0.01$, * $p < 0.05$, compared to CaOx control.)

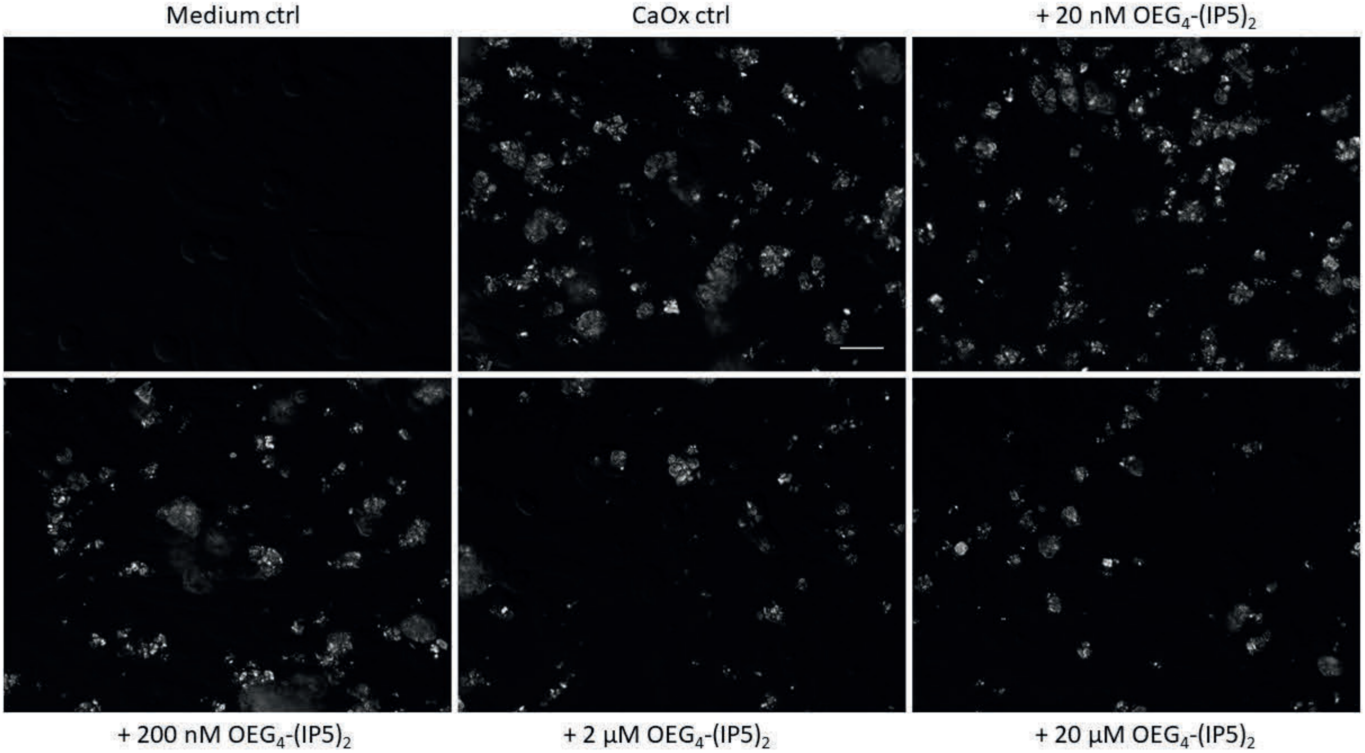

SFig.10: Efficacy of OEG$_4$-(IP5)$_2$ to remove CaOx bound to renal epithelial cells *in vitro*. RPTEC/TERT1 cells were incubated with 150 µg/cm^2 CaOx for 30 minutes, unbound CaOx was removed by washing with PBS and cells were further incubated with OEG$_4$-(IP5)$_2$ or medium for 2 h. After washing and fixation the amount of bound CaOx was measured by differential interference microscopy and quantification of the crystal occupied area. Representative images showing reduction of CaOx bound to the cell layer upon incubation with increasing doses of OEG$_4$-(IP5)$_2$ (63x oil objective, DIC mode, scale bar: 20 µm). CaOx ctrl presents cells pretreated with CaOx followed by medium incubation after washing; medium ctrl presents a cell monolayer incubated with medium only in both incubation steps.

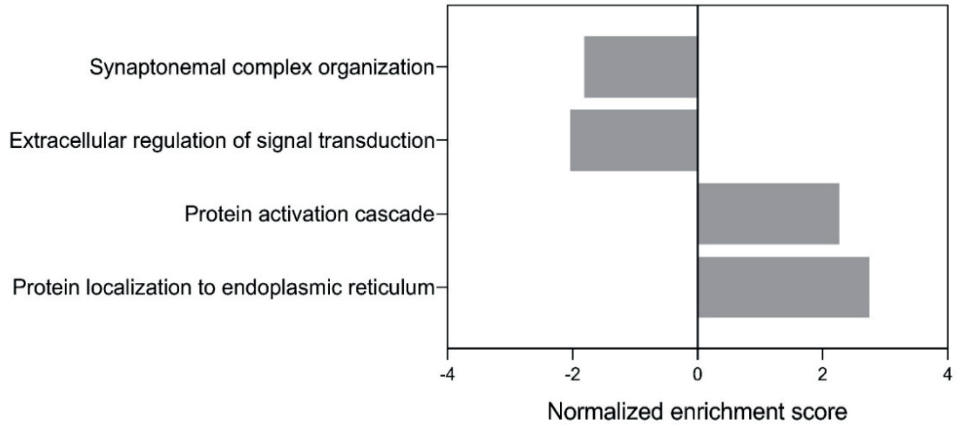

SFig.11: Gene set enrichment analysis of gene expression levels in the CaOx + OEG_4-$(IP5)_2$ relative to the medium only group. Top GO terms and corresponding normalized enrichment score for gene sets with a FDR ≤ 0.05 are shown.

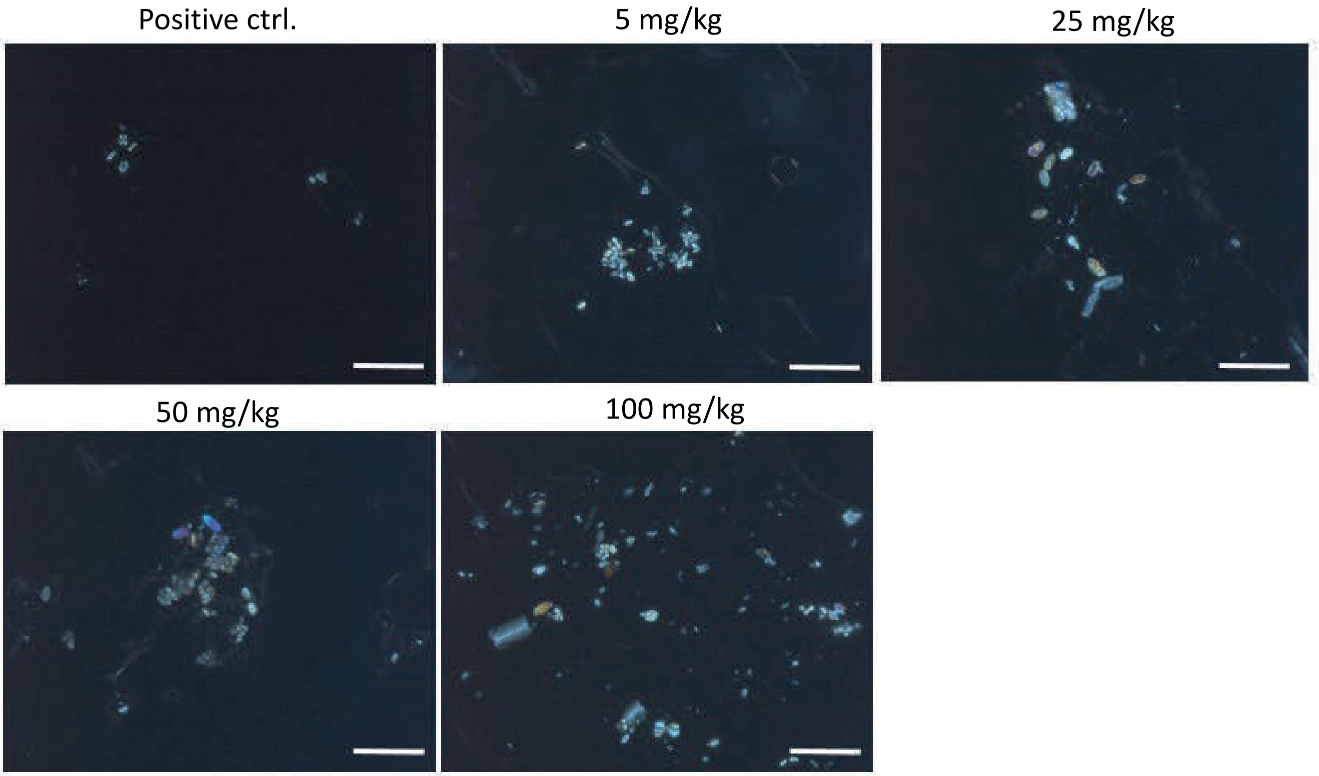

SFig.12: Urinary CaOx crystals in a mouse model of renal CaOx crystallization. C57B16/N mice were fed an oxalate enriched and calcium depleted diet to induce renal CaOx crystallization and concurrently treated with indicated concentrations of OEG_4-$(IP5)_2$ *s.c*. twice daily for 7 consecutive days. Representative images of CaOx crystals found in urine on day 7 are shown (scale bar: 200 µm). 'Pos.ctrl.' presents mice receiving the oxalate-enriched and calcium-depleted diet, and vehicle injections.

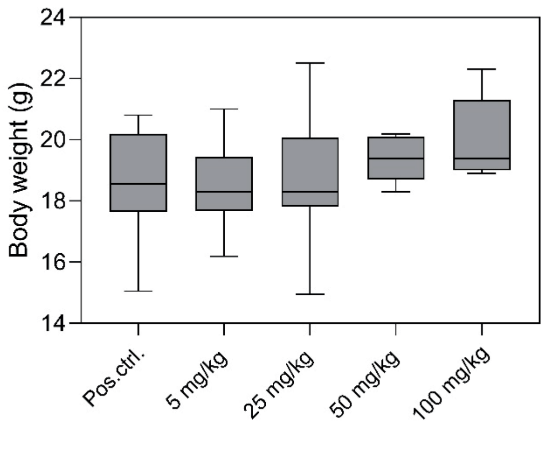

SFig.13: Body weight of mice at day 7 of treatment. C57B16/N mice were fed an oxalate enriched and calcium depleted diet to induce renal CaOx crystallization and concurrently treated with indicated concentrations of OEG_4-$(IP5)_2$ *s.c*. twice daily for 7 consecutive days. Measured body weight of mice at day 7 is plotted. Box plots show 25th to 75th percentile of data with the line expressing the median value and whiskers extending to minimum and maximum values. 'Pos.ctrl.' presents mice receiving the oxalate-enriched and calcium-depleted diet, and vehicle injections.

STable 1: Single crystal XRD analysis of CaOx crystals formed with OEG$_4$-(IP5)$_2$..

	Brightfield image	XRD structure	Crystal data and structure refinement	
Structure 1	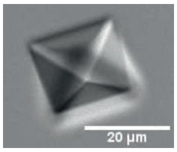	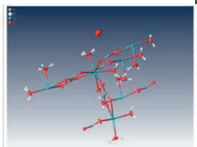	Identification code	ipw201118_1_1
			Empirical formula	$C_2H_4CaO_{6.5}$
			CSD database structure code	CAOXAL
			Formula weight	172.13
			Temperature/K	100.0(1)
			Crystal system	tetragonal
			Space group	I4/m
			a/Å	12.3356(2)
			b/Å	12.3356(2)
			c/Å	7.3293(2)
			α/°	90
			β/°	90
			γ/°	90
			Volume/Å^3	1115.28(5)
			Z	8
			ρ_{calc}g/cm^3	2.050
			μ/mm^{-1}	9.654
			F(000)	704.0
			Crystal size/mm^3	0.023 × 0.019 × 0.007
			Radiation	CuKα (λ = 1.54184)
			2Θ range for data collection/°	10.142 to 158.738
			Index ranges	$-15 \leq h \leq 13$, $-12 \leq k \leq 15$, $-8 \leq l \leq 9$
			Reflections collected	2946
			Independent reflections	641 [R_{int} = 0.0393, R_{sigma} = 0.0308]
			Data/restraints/parameters	641/5/53
			Goodness-of-fit on F^2	1.086
			Final R indexes [I>=2σ (I)]	R_1 = 0.0341, wR_2 =0.0893
			Final R indexes [all data]	R_1 = 0.0391, wR_2 =0.0919
			Largest diff. peak/hole / e Å^{-3}	0.41/-0.44
Structure 2	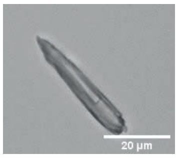	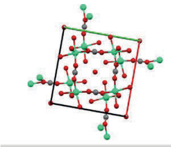	Identification code	iwp201118_2_1_a.res in I4/m
			CSD database structure code	CAOXAL
			Crystal system	tetragonal
			Space group	I4/m
			a/Å	12.3276(4)
			b/Å	12.3276(4)
			c/Å	7.3215(4)
			α/°	90
			β/°	90
			γ/°	90
			Volume/Å^3	1112.65

STable 2: Inductively coupled plasma – optical emission spectrometry analysis for phosphorus content of OEG_4-$(IP5)_2$ incubated with high calcium and oxalate concentrations in Bis Tris buffer. Assay solution was incubated for 1 h before ultrafiltration to remove CaOx particles. Filtrate was diluted 1:10 and analyzed for phosphorus content.

Sample	P (mg/L)	Experimental condition<br>Calcium (mM)	Oxalate (mM)	Compound (µM)
Pos.crtl	0.166	0	0	10
Compound-CaOx	<loq	5	5	10
Neg.ctrl	<loq	5	5	0

STable 3: OEG_4-$(IP5)_2$ reduces renal CaOx deposition and injury in a mouse model of renal CaOx crystallization. Statistical analysis of dose-dependent effects of OEG_4-$(IP5)_2$ on renal CaOx deposition, tubular injury, plasma BUN and urinary oxalate levels. Normality of data was assessed by D'Agostino and Pearson testing and found to be normally distributed. Ordinary one-way ANOVA followed by post-hoc Dunnett's multiple comparison was performed. Adjusted p-value is given. 'Vehicle control' presents mice receiving the oxalate-enriched and calcium-depleted diet, and vehicle injections; 'Negative control' includes healthy mice not receiving the oxalate-enriched and calcium-depleted diet and no injections.

Groups	**CaOx area**	**Tubular injury score**	**Plasma BUN**	**Urinary oxalate**
Vehicle control *vs.* negative control	<0.0001	<0,0001	0.0128	0.0552
Vehicle control *vs.* 5 mg/kg	0.0028	0.9747	0.5131	0.0066
Vehicle control *vs.* 25 mg/kg	0.0002	0.2758	0.9803	0.0210
Vehicle control *vs.* 50 mg/kg	<0.0001	0.0009	0.052	0.0156
Vehicle control *vs.* 100 mg/kg	<0.0001	<0,0001	0.0272	0.0013
